# Mutations That Alter the Bacterial Cell Envelope Increase Lipid Production

**DOI:** 10.1128/mBio.00513-17

**Published:** 2017-05-23

**Authors:** Kimberly C. Lemmer, Weiping Zhang, Samantha J. Langer, Alice C. Dohnalkova, Dehong Hu, Rachelle A. Lemke, Jeff S. Piotrowski, Galya Orr, Daniel R. Noguera, Timothy J. Donohue

**Affiliations:** a DOE Great Lakes Bioenergy Research Center, University of Wisconsin—Madison, Madison, Wisconsin, USA; b Department of Bacteriology, University of Wisconsin—Madison, Madison, Wisconsin, USA; c Department of Civil and Environmental Engineering, University of Wisconsin—Madison, Madison, Wisconsin, USA; d Pacific Northwest National Laboratory, Environmental Molecular Sciences Laboratory, Richland, Washington, USA; University of Hawaii at Manoa

**Keywords:** *Rhodobacter*, bioreactors, cell envelope, fatty acids, lipid synthesis, two-component regulatory systems

## Abstract

Lipids from microbes offer a promising source of renewable alternatives to petroleum-derived compounds. In particular, oleaginous microbes are of interest because they accumulate a large fraction of their biomass as lipids. In this study, we analyzed genetic changes that alter lipid accumulation in *Rhodobacter sphaeroides*. By screening an *R. sphaeroides* Tn*5* mutant library for insertions that increased fatty acid content, we identified 10 high-lipid (HL) mutants for further characterization. These HL mutants exhibited increased sensitivity to drugs that target the bacterial cell envelope and changes in shape, and some had the ability to secrete lipids, with two HL mutants accumulating ~60% of their total lipids extracellularly. When one of the highest-lipid-secreting strains was grown in a fed-batch bioreactor, its lipid content was comparable to that of oleaginous microbes, with the majority of the lipids secreted into the medium. Based on the properties of these HL mutants, we conclude that alterations of the cell envelope are a previously unreported approach to increase microbial lipid production. We also propose that this approach may be combined with knowledge about biosynthetic pathways, in this or other microbes, to increase production of lipids and other chemicals.

## IMPORTANCE

This paper reports on experiments to understand how to increase microbial lipid production. Microbial lipids are often cited as one renewable replacement for petroleum-based fuels and chemicals, but strategies to increase the yield of these compounds are needed to achieve this goal. While lipid biosynthesis is often well understood, increasing yields of these compounds to industrially relevant levels is a challenge, especially since genetic, synthetic biology, or engineering approaches are not feasible in many microbes. We show that altering the bacterial cell envelope can be used to increase microbial lipid production. We also find that the utility of some of these alterations can be enhanced by growing cells in bioreactor configurations that can be used industrially. We propose that our findings can inform current and future efforts to increase production of microbial lipids, other fuels, or chemicals that are currently derived from petroleum.

## INTRODUCTION

The recent advances in genome, systems, and synthetic biology, when coupled with the diverse metabolic activities of microbes, provide an opportunity to produce valuable compounds from renewable resources. Lipids derived from microbes offer a promising source of renewable fuels and chemicals, to offset petroleum usage and reduce CO_2_ emissions (1, 2). A major challenge to producing microbial replacements for oils that are cost-competitive with petroleum products is increasing the yield of these lipids, which are energetically expensive for cells to produce and thus tightly regulated (3, 4). One approach for microbial oil production is the use of oleaginous microbes, defined as those accumulating over 20% of their dry cell weight (DCW) as lipid (1). However, even though very high oil content (up to 90%) can be observed under some experimental conditions, lipid content is usually not high under nutrient-replete conditions (4, 5). Genetic and process engineering strategies are being investigated to further increase the biomass lipid content and yield of oleaginous microbes (1, 5). However, although biosynthetic pathways for fatty acids and lipids are well understood in some microbes, identifying and bypassing the mechanisms regulating lipid accumulation in oleaginous strains remain a challenge (1, 4, 6).

An alternative approach to increase production of lipids in microbes is transgenic engineering of lipogenic pathways into nonoleaginous but robust and genetically tractable hosts (7). Many enzymes that convert fatty acids, or pathway intermediates, into products with desirable fuel properties have been investigated (8–13). However, achieving industrially relevant lipid production levels and yields can require genetic and metabolic engineering steps that are not feasible in many hosts (7).

This paper reports on experiments to acquire new knowledge about how microbes control lipid production. To do this, we study *Rhodobacter sphaeroides*, a facultative purple nonsulfur bacterium. Unlike the case with many well-studied facultative bacteria, changes in O_2_ tension cause significant morphological changes in the cell envelope of this Gram-negative bacterium (14–16). In response to low O_2_ tension, *R. sphaeroides* increases its intracellular membrane surface area, developing specialized intracytoplasmic membrane (ICM) invaginations that protrude into the cytoplasm (14–16). This cell envelope remodeling under low-O_2_ conditions increases the cellular phospholipid content ~3-fold (17). Thus, we sought to gain an increased understanding of the native ability of *R. sphaeroides* to regulate lipid content.

To do this, we screened for *R. sphaeroides* mutants with increased lipid production. Many of these high-lipid (HL) mutants were found to have increased sensitivity to drugs that target the cell envelope and have altered cell shape, and several of them secreted cellular lipids. When we grew one of the highest-lipid-secreting strains in a fed-batch bioreactor, fatty acids represented 33% of the DCW of this culture. Thus, by genetically altering cell envelope functions we can increase lipid production and have identified some mutations that convert *R. sphaeroides* into an oleaginous bacterium. We propose that changes in cell surface or envelope functions can be used to increase production of lipids and additional bioproducts in other microbes.

## RESULTS

### Identification of HL mutants.

We sought to leverage the native ability of *R. sphaeroides* to alter its fatty acid content (17) in order to understand systems that control bacterial lipid accumulation. Our approach was to identify mutant strains that had increased levels of fatty acid at high O_2_ when cells normally lack an ICM (14–16) and have a lower lipid content (17). We chose not to screen for mutants that have low lipid levels at reduced O_2_ tensions because previous analyses have shown that such mutations would likely interfere with ICM production for other reasons (17–19). We used a parent strain (Δ0382) that is unable to make the hydrophobic polymer polyhydroxybutyrate (PHB) (20) so that we could use the fluorescence intensity of Nile red-stained cells as a proxy of lipid content.

**FIG 1:**
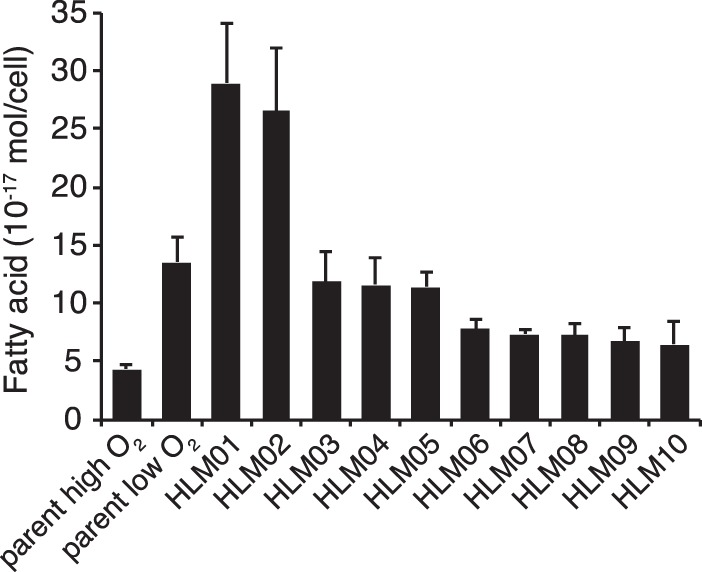
Fatty acid content of parent strain grown at high and low O_2_ compared to high-lipid (HL) mutants grown at high O_2_. Data shown represent the means from three or more independent cultures ± standard deviations.

To identify potential high-lipid (HL) mutants, we screened a library of ~11,400 strains generated by Tn*5* transposon mutagenesis. Previous genetic analysis of *R. sphaeroides* indicates that a library containing ~10^4^ insertions is sufficient to obtain a representative set of mutants (21). When the fatty acid content of the top 30 Nile red-staining mutants was quantified by gas chromatography-mass spectrometry (GC-MS), we found a set of 10 unique strains (named HLM01 to HLM10) that had a ≥1.5-fold increase in fatty acid content per cell when grown at high O_2_ (Fig. 1). Two mutants (HLM01 and HLM02) had an ~6-fold increase in fatty acids over the parent strain grown at high O_2_ (Fig. 1), an increase twice that observed when the parent strain was grown at low O_2_ (Fig. 1).

### Genes and processes disrupted in HL mutants.

The transposon insertion sites identified in these 10 HL mutants (Table 1) did not reveal disruption of genes typically targeted for increasing lipid accumulation, such as those for central carbon metabolism or fatty acid biosynthesis and degradation (22, 23). Instead, the genes inactivated in the HL mutants encoded a diverse group of proteins, including a transcription factor, a chaperone, proteases, and putative secreted and cell envelope proteins.

**TABLE 1:** Transposon insertion sites in HL mutants^a^

Strain	FA inc. (fold)	Insertion site	ORF(s) disrupted, with annotation	Sig. pep.	TM helix
HLM01	6.7	Chr1: 1471645	RSP2839, NtrY sensor signal transduction histidine kinase	No	5
			RSP2840, NtrX response regulator	No	None
HLM02	6.1	Chr1: 1469665	RSP2840, NtrX response regulator	No	None
HLM03	2.7	Chr2: 274987	RSP3218, cob(II)yrinic acid a,c-diamide reductase/5,6-dimethylbenzimidazole synthase	No	None
HLM04	2.7	Chr1: 2814885	RSP1056, signal transduction histidine kinase	No	2
HLM05	2.6	Chr1: 2970757	RSP1200, uncharacterized conserved protein YkwD	Yes	None
HLM06	1.8	Chr2: 938456	RSP1422, chromosome partitioning protein, ParB family	No	None
HLM07	1.7	Chr1: 2086261	RSP0355, periplasmic serine protease DegP	No	1
HLM08	1.7	Chr1: 1189239	RSP2545, stationary-phase survival protein SurE	No	None
			RSP2544, protein-l-isoaspartate *O*-methyltransferase (pcm)	No	None
			RSP2543, peptidoglycan dd-endopeptidase	Yes	None
HLM09	1.5	Chr1: 1395725	RSP2745, Stealth protein	No	None
HLM10	1.5	Chr1: 916649	RSP2293, ClpA, ATP-dependent Clp protease ATP-binding subunit	No	None

^a^ Strains are sorted from highest to lowest fold increase in total fatty acid (FA inc.) compared to the parent strain. The presence of a signal peptide (Sig. pep.) was predicted by SignalP 4.1 (http://www.cbs.dtu.dk/services/SignalP/), and the number of predicted transmembrane helixes (TM helix) was determined by TMHMM Server v.2.0 (http://www.cbs.dtu.dk/services/TMHMM/).

To gain insight into how the disrupted genes products affect lipid content, we used chemical sensitivity analysis to characterize what cellular processes were affected in the HL mutants (24). To do this, we tested the impact on growth of a set of compounds that affect protein synthesis, folic acid biosynthesis, membrane integrity, peptidoglycan biosynthesis, and DNA integrity (see Table S2 in the supplemental material). For some compounds, such as the protein synthesis inhibitor neomycin, we saw no growth difference between the HL mutants and the parent strain (Fig. S1A). For other compounds, including the detergent sodium dodecyl sulfate (SDS), many or all of the HL mutants showed increased sensitivity (Fig. S1B), while for others, such as the peptidoglycan-active antibiotic amoxicillin, we saw increased sensitivity in one or more HL mutants compared to the parent strain (Fig. S1C). By analyzing the relative growth of all the HL mutants treated with compounds having common cellular targets, we observed that these strains were most sensitive to compounds active on the cell or outer membranes (OMs) (62% of the parent cell growth).

10.1128/mBio.00513-17.1 FIG S1  Relative fitness of HL mutants in the presence of indicated compounds compared to the parent strain. Data shown represent the means from three cultures ± standard deviations. Download FIG S1, PDF file, 0.2 MB. Copyright © 2017 Lemmer et al. 2017 Lemmer et al. This content is distributed under the terms of the Creative Commons Attribution 4.0 International license.

**FIG 2:**
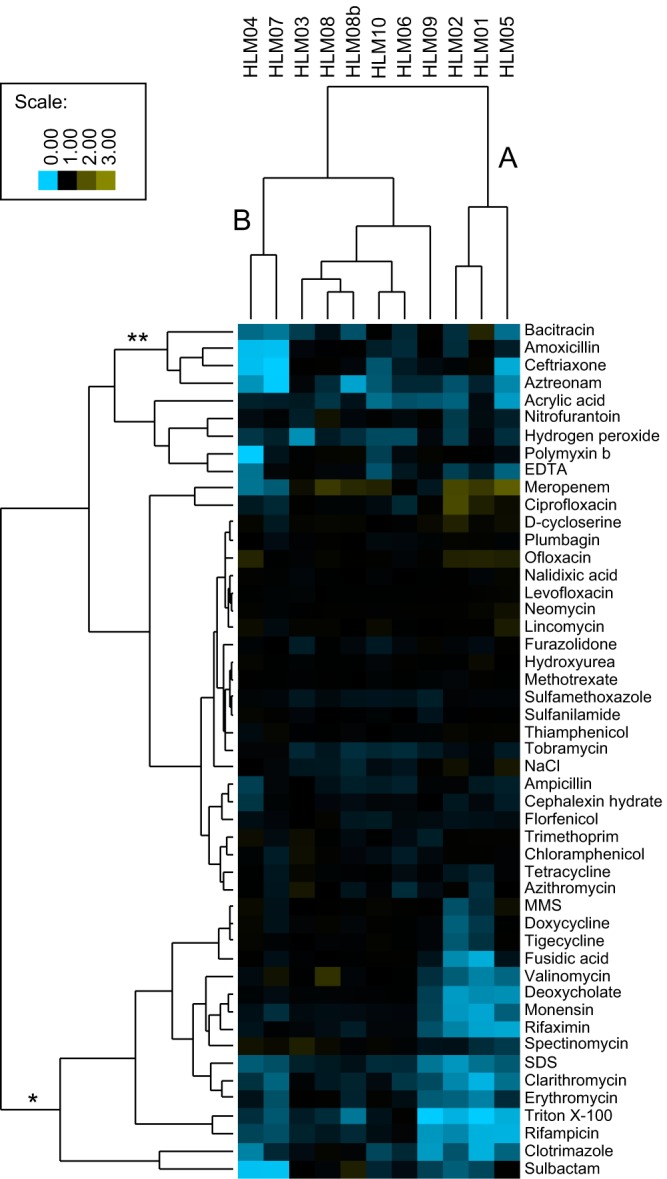
Chemical sensitivity analysis of HL mutants. Cluster A shows increased sensitivity to a group of compounds that report on membrane integrity (*). Cluster B shows increased sensitivity to a group of peptidoglycan active compounds (**). The color scale indicates relative fitness compared to the parent strain. A value of 1 (black) indicates no change relative to the parent, <1 (blue) indicates increased sensitivity to the compound, and >1 (yellow) indicates increased resistance to the compound. MMS, methyl methanesulfonate.

Clustering of the mutants and the compounds based on relative growth in the presence of these chemicals (Fig. 2) showed that strains HLM01, HLM02, and HLM05 formed a cluster separate from the other strains (labeled A in Fig. 2). Two of these strains, HLM01 and HLM02, have mutations in genes that are predicted to act in the same pathway (the NtrYX two-component system [Table 1]), and so it is not surprising that they behave similarly in this analysis. The third strain in this cluster (HLM05) has a mutation in a conserved uncharacterized membrane protein (see Discussion). These three HL mutants are sensitive to a cluster of compounds (marked * in Fig. 2) containing membrane-targeting detergents and ionophores, as well as the RNA polymerase inhibitors rifampin and rifaximin and the protein synthesis inhibitors erythromycin and clarithromycin. The latter 4 compounds do not target the membrane, but it is known that decreased membrane integrity can sensitize cells to these hydrophobic drugs (25). Thus, these three HL mutants share increased sensitivity to compounds that are associated with decreased membrane integrity.

A second cluster of HL mutants (HLM04 and HLM07, labeled B in Fig. 2) share increased sensitivity to a group of compounds that inhibit peptidoglycan biosynthesis (amoxicillin, aztreonam, bacitracin, and ceftriaxone, marked ** in Fig. 2). This suggests that the mutations in these two HL strains alter the integrity of the peptidoglycan cell wall.

The isolation of HL mutants with sensitivities to different classes of bioactive compounds suggests that there may be multiple mechanisms causing increased lipid production. While the HL mutants HLM03, HLM06, and HLM08 to HLM10 showed increased sensitivity to some other compounds (e.g., hydrogen peroxide for HLM03), these data did not support predictions of specific processes that might be impaired in these strains. In sum, the chemical sensitivity analysis showed that many of the HL mutants had increased sensitivity to compounds that act at the cell envelope, either at the membrane or at the cell wall.

### Morphological changes in HL mutants.

**FIG 3:**
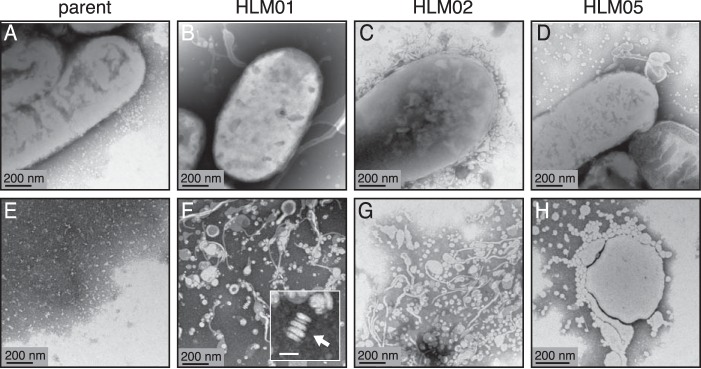
TEM of whole mounts of the parent strain (A and E) and HL mutants (B to D and F to H). The bottom row of panels (E to H) shows views of extracellular material from these strains. Similar micrographs of the parent strain and other HL mutants are shown in Fig. S2 and S3 in the supplemental material. The arrow in the inset (F) indicates a stacked structure typical of liposomes; bar for this inset panel, 50 nm.

Based on the above finding, we used transmission electron microscopy (TEM) of whole-mount cells to assess morphological changes in the cell envelope of the HL strains. This analysis revealed that those HL mutants which are sensitive to membrane-active compounds (cluster A; HLM01, HLM02, and HLM05) produced extracellular material adjacent to (Fig. 3B to D) and separated from (Fig. 3F to H) the cells that was not seen in the parent strain (Fig. 3A and E). Samples of HLM05 had round extracellular structures in the range of 20 to 50 nm (Fig. 3H), while samples from HLM01 and HLM02 contained round and irregular structures, as well as some stacked structures that are often observed when liposomes are in aqueous solution (Fig. 3F and G) (26). Another HL mutant (HLM08) produced extracellular material, some of which was organized in stacked structures (Fig. S2A and B). Two other HL mutants (HLM03 and HLM09) lacked a significant amount of extracellular material but instead had structures that appeared to derive from and adhere to the cell surface (Fig. S2C and D). The membrane protrusions and secretions seen by TEM analysis of HLM01 to HLM03, HLM05, HLM08, and HLM09 are consistent with alterations in the cell envelope predicted for some of these strains by chemical sensitivity analysis.

10.1128/mBio.00513-17.2 FIG S2  TEM micrographs of whole mounts of indicated HL mutants. (A and B) HLM08 has extracellular material and irregularly shaped particles of varied sizes as well as stacked structures. (C and D) HLM03 (C) and HLM09 (D) have structures extending off the cell surface (arrows). Download FIG S2, PDF file, 12.6 MB. Copyright © 2017 Lemmer et al. 2017 Lemmer et al. This content is distributed under the terms of the Creative Commons Attribution 4.0 International license.

TEM of the two HL mutants that are sensitive to cell wall-active compounds (cluster B; HLM04 and HLM07) did not show evidence of extracellular material but instead suggested that they had a different shape than the parent strain (Fig. S3A to C). Measurement of cell dimensions of HLM04 and HLM07 by superresolution structured illumination microscopy (SIM) (Fig. S3D to F and S4) showed that they were shorter than the parent cells but that the cell width was similar to that of the parent (Table 2). Overall, SIM analysis showed that 8 out of the 10 HL mutants had differences in cell length and/or width from the parent strain (Table 2), providing additional support for the hypothesis that changes in the cell envelope were a common feature among many of these strains.

10.1128/mBio.00513-17.3 FIG S3  Cell shape of HL mutants HLM04 and HLM07 compared to the parent strain. (A to C) TEM of cell whole mounts. (D to F) Superresolution structured illumination microscopy (SIM) of Nile red-stained cells. Bars (D to F), 1 µm. Download FIG S3, PDF file, 9 MB. Copyright © 2017 Lemmer et al. 2017 Lemmer et al. This content is distributed under the terms of the Creative Commons Attribution 4.0 International license.

10.1128/mBio.00513-17.4 FIG S4  SIM images of Nile red-stained parent and HL mutant cells. Representative fields are shown from images used to measure cell dimensions. Bar, 2 µm. Download FIG S4, PDF file, 1.2 MB. Copyright © 2017 Lemmer et al. 2017 Lemmer et al. This content is distributed under the terms of the Creative Commons Attribution 4.0 International license.

**TABLE 2:** Measurements of cell length and width of Nile red-stained cells by superresolution fluorescence microscopy^a^

Strain	Length (μm)	Width (μm)	*n*	Difference(s)
Parent	1.72 ± 0.38	0.72 ± 0.05	75	
HLM01	1.70 ± 0.41	0.76 ± 0.06*	259	Wider
HLM02	1.68 ± 0.46	0.73 ± 0.05	205	
HLM03	2.32 ± 0.51*	0.71 ± 0.05	91	Longer
HLM04	1.22 ± 0.22*	0.73 ± 0.05	148	Shorter
HLM05	2.36 ± 0.49*	0.75 ± 0.06**	111	Longer and wider
HLM06	1.79 ± 0.36	0.73 ± 0.06	88	
HLM07	1.41 ± 0.27*	0.73 ± 0.06	86	Shorter
HLM08	1.73 ± 0.33	0.67 ± 0.06*	102	Narrower
HLM09	1.83 ± 0.46	0.70 ± 0.05**	126	Narrower
HLM10	1.60 ± 0.30***	0.74 ± 0.06	104	Shorter
Parent with low O_2_	2.27 ± 0.72*	0.83 ± 0.08*	83	Longer and wider

^a^ Measurements are expressed as means ± standard deviations, with *n* being number of cells measured. Significant differences from the parent strain are indicated as follows: *, *P* < 0.0001; **, *P* < 0.002; ***, *P* < 0.03.

### Lipid secretion by HL mutants.

**FIG 4:**
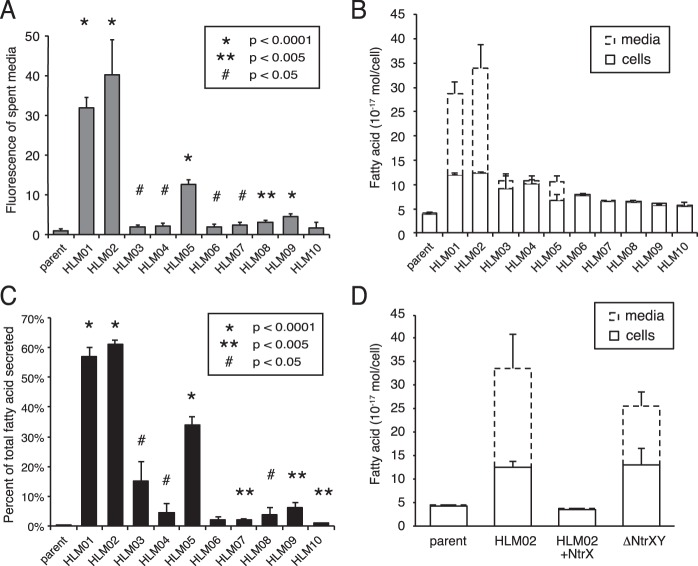
Analysis of the extracellular material of parent and HL mutant strains. (A) Nile red staining of the media from parent and HL mutant cultures. (B) Fatty acid content of parent and HL mutant cultures separated into cell and medium fractions, shown as a stacked bar graph. (C) Percentage of total fatty acids found in the medium fraction for data shown in panel B. (D) Fatty acid content of cell and medium fractions for parent and HLM02 strains compared to HLM02 with a plasmid expressing NtrX (HLM02 + NtrX) and the parent strain with a deletion of *ntrY* and *ntrX* (ΔNtrYX), shown as a stacked bar graph. Data shown represent the means from three or more independent cultures ± standard deviations. *P* values are for the difference of each HL mutant from the parent strain.

Since we observed materials on the surface or the outside of the HL mutants by TEM, we stained the medium with Nile red to test for the presence of hydrophobic compounds. We found that media from all but one of the HL mutants had increased Nile red staining compared to that from the parent strain (Fig. 4A). In particular, cluster A strains (HLM01, HLM02, and HLM05) that had the largest amount of extracellular material by TEM had 13- to 40-fold increases in fluorescence over that of the parent strain (Fig. 4A).

We tested if the increased Nile red staining of the HL mutants was due to the presence of extracellular lipid by quantifying fatty acid levels in culture supernatants. For the parent strain, a low level (0.2%) of the fatty acid in the total culture (cells plus supernatant) was present in the medium (Fig. 4B and C), likely representing incomplete separation of cells and medium. In contrast, 9 of the 10 HL mutants had a statistically significant increase in fatty acid in the medium compared to the parent strain. Consistently with the large amount of extracellular material observed by TEM, strains HLM01, HLM02, and HLM05 had the highest percentage (≥35%) of the total fatty acid present in the medium (Fig. 4B and C).

To further characterize the secreted material, we analyzed fatty acid and lipid phosphorus levels in the culture supernatants of HLM01, HLM02, and HLM05. For all three of these HL mutants, the fatty acid-to-lipid phosphorus ratio of the supernatants was 1.5, close to the 2:1 ratio expected for phospholipid (Fig. S5A). We also quantified lipopolysaccharide (LPS) in the supernatants and found that LPS-associated fatty acids accounted for less than 1% of the secreted fatty acid for HLM01 and HLM02 (Fig. S5B). Therefore, we conclude that the secreted lipid is composed primarily of phospholipid.

10.1128/mBio.00513-17.5 FIG S5  Analysis of extracellular material of HLM01, HLM02, and HLM05 for phospholipid and LPS content. (A) Fatty acid and lipid phosphorus levels in the medium with molar ratios listed below the graph. (B) LPS content of the medium, with conversion to estimated LPS-associated fatty acids on the secondary vertical axis. The relative contribution of LPS-associated fatty acids to the total amount of secreted fatty acids is shown below the graph. Data shown represent the means from four or more independent cultures ± standard deviations. Download FIG S5, PDF file, 0.2 MB. Copyright © 2017 Lemmer et al. 2017 Lemmer et al. This content is distributed under the terms of the Creative Commons Attribution 4.0 International license.

The two mutants with the highest levels of lipid secretions, HLM01 and HLM02, both have insertions in genes encoding components of the NtrYX two-component system (Table 1). To verify that disruption of these genes was causal of the HL phenotype, we showed that fatty acid levels and secretion were restored to wild-type levels by complementation with a plasmid expressing NtrX (Fig. 4D). We also showed that deleting *ntrX* and *ntrY* from the parent strain (ΔNtrYXpd) caused a similar increase in fatty acid content and secretion as that seen in HLM01 and HLM02 (Fig. 4D).

### Fatty acid productivity of an HL-secreting strain.

Extracellular lipid accumulation by some HL mutants could make them attractive for production of biofuels or bioproducts. To assess fatty acid production, we chose HLM02 for further study since it is one of two HL mutants with the highest level of extracellular lipid.

**FIG 5:**
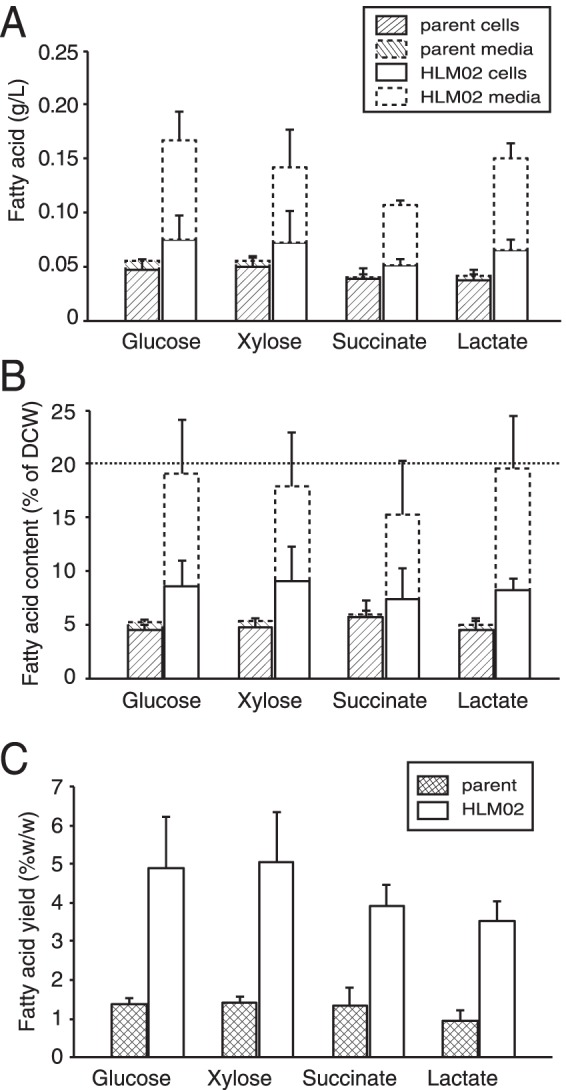
Fatty acid production by parent and HLM02 strains in batch cultures with one of four different carbon sources. (A and B) Fatty acid productivity per culture volume (A) and fatty acid content as a percentage of dry cell weight (DCW) (B). For each condition, the cell and medium fractions are stacked. (C) Fatty acid yield per carbon substrate consumed. Data shown represent the means from three or more independent cultures ± standard deviations.

When we compared the fatty acid productivity (grams fatty acid per liter) of HLM02 cultures to that of the parent strain, they produced similar amounts of intracellular lipid (Fig. 5A), when grown in batch culture with succinate as a carbon source. However, if one includes cellular and secreted lipids, total fatty acid productivity was 2.7-fold higher in the HLM02 culture than in the parent strain (Fig. 5A) (*P* < 0.001). This increase in total fatty acid productivity for HLM02 is smaller than the increase observed when measuring fatty acid content per cell (Fig. 1) because HLM02 does not achieve as high a cell density in batch culture as does the parent strain.

*R. sphaeroides* can metabolize a wide variety of carbon substrates (27), and so we also tested fatty acid productivity in cultures containing a different organic acid (lactate, which is a common fermentation by-product [28]), as well as sugars (glucose and xylose, which are abundant in cellulosic biomass hydrolysates [29]). Fatty acid productivity was increased in HLM02 compared to the parent strain when using each of these carbon sources (Fig. 5A), with ~50 to 55% of the total fatty acid found in the culture supernatant. For each of the carbon sources tested, the cellular fatty acids represented 5 to 6% of the dry cell weight (DCW) in the parent and HLM02 strain. However, when the secreted lipid was included in the calculations, the total fatty acid content of HLM02 was 15 to 20% of the DCW (Fig. 5B).

Another common metric for analysis of production strains is product yield per amount of carbon source consumed. For the parent strain, the total fatty acid yield from each of the carbon sources tested was 1.0 to 1.4% (wt/wt) (Fig. 5C). For the HLM02 mutant, the fatty acid yield increased 2.9- to 3.7-fold (*P* ≤ 0.01 for all substrates) to 3.5 to 5.0% (wt/wt) (Fig. 5C). There was no significant difference in fatty acid yield of HLM02 between the different carbon sources tested. The maximum theoretical yield, if all of the carbon substrate were converted into fatty acids by *R. sphaeroides*, is ~35% for glucose, xylose, and succinate and ~28% percent for lactate. Thus, the total fatty acid yields (cellular plus secreted) for batch cultures of HLM02 were 11 to 14% of the theoretical yield on these carbon sources.

### Extracellular production of novel fatty acids.

The utility of a lipid-secreting mutant would be increased if it also produced novel fatty acids extracellularly. *R. sphaeroides* has recently been reported to make a furan-containing fatty acid, 10,13-epoxy-11-methyl-octadecadienoic acid (19Fu-FA), which has several potential uses (30). Elevated levels of 19Fu-FA are found in a mutant that lacks the ChrR anti-sigma factor (30). In order to test if *R. sphaeroides* could secrete 19Fu-FA, we deleted *chrR* from the ΔNtrYXpd strain (ΔChrRΔNtrYXpd). We found that 19Fu-FA was ~3 to 4% of the total fatty acids in both the cellular and the supernatant fractions of the ΔChrRΔNtrYXpd strain (Table S3). From this, we conclude that the HL mutants can also secrete novel fatty acids into the medium.

### Increasing fatty acid production of an HL mutant by high-density growth.

Given the extracellular production of lipids by some HL mutants, we wanted to test if the productivity of these strains could be increased by the use of high-density cultures. We reasoned that, if product (fatty acid) formation is tied to cell number, then increasing culture density should increase the culture productivity. We opted to use a fed-batch bioreactor (31–33) to test this hypothesis, since it can bypass the negative impacts of high (toxicity) or low (limitation) nutrient availability.

In these experiments, we used real-time measurement of reactor dissolved O_2_ (DO) as an indicator of substrate limitation (34), since decreased cellular respiration should cause an increase in DO. This is illustrated for an *R. sphaeroides* xylose-fed culture in Fig. S6: when the DO increases, nutrients are provided to the reactor, causing the DO to decrease again, presumably when cellular respiration increases. This feeding cycle is repeated iteratively throughout the reactor run in order to obtain high-density cultures.

10.1128/mBio.00513-17.6 FIG S6  Feeding protocol for fed-batch high-density cultures. The solid line shows culture dissolved oxygen (DO), which is maintained at a low baseline level by bubbling the reactor with saturated air. Increases in DO (seen at ~51.2 and 52.6 h) indicate reduced metabolic activity in the reactor, and at these times, a bolus of feeding medium is added, increasing the total feeding volume (dotted line). After feeding, the culture DO drops to baseline as cellular respiration increases. A 3-h period is shown from a 120-h reactor run; the illustrated process repeats iteratively throughout the reactor incubation. Download FIG S6, PDF file, 0.1 MB. Copyright © 2017 Lemmer et al. 2017 Lemmer et al. This content is distributed under the terms of the Creative Commons Attribution 4.0 International license.

**FIG 6:**
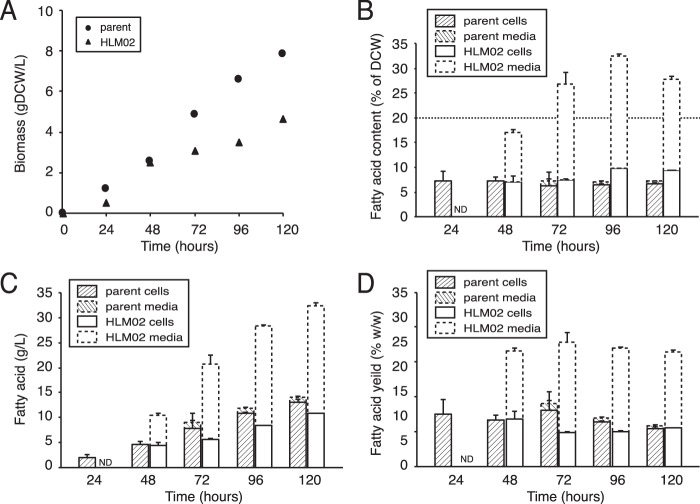
Fed-batch reactor production of fatty acids by HLM02 mutant compared to the parent strain, grown with xylose as a carbon source. (A and B) Total biomass (A) and fatty acid content (B) in the fed-batch reactor. (C) Productivity of fatty acids. (D) Yield of fatty acids per xylose consumed. Data are shown from a representative bioreactor run for each strain; cell and medium fractions are stacked; error bars represent standard deviations between technical replicates.

When cells were grown in a fed-batch reactor using this feeding regimen and xylose as a carbon source, cell density increased for ~120 h and then plateaued. Under these conditions, the parent strain culture reached a maximal density of 7.9 g DCW/liter (Fig. 6A), which had increased from 1.0 g DCW/liter in xylose batch culture, and the fatty acid content was stable at ~7% of DCW (Fig. 6B), comparable to the ~5% observed in a xylose batch culture (Fig. 5B). This small increase in fatty acid content of the parent strain likely reflects lower O_2_ tension in the fed-batch reactor than in the batch cultures. However, growth in the fed-batch reactor increased total fatty acid productivity 10-fold, from 0.05 g/liter in batch culture (Fig. 5A) to 0.50 g/liter (Fig. 6C). In addition, the fatty acid yield from xylose increased from 1.4% (wt/wt) in the batch culture (Fig. 5C) to a maximum of 3.6% in the fed-batch reactor (Fig. 6D).

To analyze the utility of using fed-batch reactors to increase product yield, we again used HLM02 since this was one of the two HL mutants with the highest productivity in batch culture (see above). Under identical conditions, the HLM02 mutant grew at a lower rate and to lower final cell density than the parent in the fed-batch reactor (4.7 g DCW/liter, an ~6-fold increase from 0.8 g DCW/liter in a xylose-fed batch culture) (Fig. 6A). Despite this, HLM02 produced more than twice as much total fatty acids as the parent strain (~1.3 g/liter at 120 h [Fig. 6C]), which is ~9 times the amount of fatty acid produced by HLM02 in batch culture. This demonstrates that growth in a fed-batch bioreactor can be used to increase fatty acid productivity by some HL mutants, even though there are differences in growth rate and total biomass production between this and a parent strain. We also found that the fatty acid content of the HLM02 mutant increased over time, reaching ~33% of DCW at 100 h (Fig. 6B). This represents an ~5-fold increase in fatty acid content of HLM02 compared to the parent strain under the same conditions and an 85% increase compared to the fatty acid content of HLM02 grown on xylose in a batch culture. In addition, a higher percentage of the fatty acids produced by HLM02 were secreted in the fed-batch reactor than in the batch culture (up to 69% of the total fatty acids [Fig. 6C]). When these cells were examined by TEM, we observed extracellular structures around and away from the cells (Fig. S7), at a level that appears higher than that of the same strain grown in batch culture (Fig. 3C and F).

10.1128/mBio.00513-17.7 FIG S7  TEM of whole mounts of the HLM02 mutant grown in the fed-batch bioreactor. Views are shown of cells surrounded by extracellular structures (A and B) and of extracellular structures only (C and D). Download FIG S7, PDF file, 13.6 MB. Copyright © 2017 Lemmer et al. 2017 Lemmer et al. This content is distributed under the terms of the Creative Commons Attribution 4.0 International license.

Finally, we found that fatty acid yield per xylose consumed (percent [wt/wt]) in the fed-batch bioreactor was more than doubled in HLM02 compared to the parent strain at all time points tested (Fig. 6D). The maximum fatty acid yield observed for HLM02 in the fed batch reactor (8.4%) represents ~24% of the maximum theoretical yield from cells using xylose as a carbon source (35% [see above]).

## DISCUSSION

The diverse lifestyles and metabolic activities of microbes make them attractive hosts for the production of fuels, chemicals, and other compounds. In order to achieve high-enough yields to be cost-competitive alternatives, a combination of metabolic engineering and process optimization, often tailored to the organism and product, is necessary.

Microbial lipids are an example of molecules that have advantages for use as fuels or chemicals that are currently derived from petroleum (7). In this study, we took a unique approach to understanding how to increase accumulation of microbial lipids. We sought to use a well-studied bacterium, *R. sphaeroides*, which has a native ability to increase its lipid content, to increase the synthesis of lipids. We identified a set of mutations that increase the lipid content of *R. sphaeroides*, with several of these leading to the secretion of lipids and one HL mutant producing lipids at levels found in oleaginous microbes.

### Properties of the HL mutants.

The HL mutants that we identified had increased sensitivity to cell wall- and membrane-targeting drugs, changes in cell shape, outer membrane (OM) protrusions, and secreted lipids. These phenotypes suggest that many of the HL mutants have cell envelope alterations which in some strains lead to release of cellular lipids. It is noteworthy that increased lipid production by wild-type *R. sphaeroides* involves changes in the cell envelope: cells increase their inner membrane surface area by creating ICM vesicles that protrude into the cytoplasm (15). We are not aware of other reports of changes in the cell envelope leading to increased cellular lipid content. It is also unknown whether any genes disrupted in the HL mutants play a role in assembly of the ICM, which normally occurs at low O_2_ tensions in this bacterium.

### Genetic links of HL mutants to the cell envelope.

While none of the genes disrupted in the HL mutants have previously been studied in *R. sphaeroides*, many of them had predicted functions associated with the cell envelope. RSP0355 (inactivated in HLM07) encodes one of several periplasmic serine protease (DegP) homologues in this bacterium, a protein that in other bacteria functions in protein quality control, degrading misfolded periplasmic proteins (35). RSP2543 (inactivated in HLM08) encodes a putative periplasmic cell wall hydrolase that contains a signal peptide (Table 1), a LysM peptidoglycan binding motif (36), and a Gly-Gly endopeptidase domain, suggesting that this protein plays a previously unreported role in peptidoglycan remodeling. RSP2745 (inactivated in HLM09) is a putative Stealth family protein (37), which in bacteria can function in exopolysaccharide synthesis (37). Finally, the gene inactivated in HLM10 (RSP2293) encodes the ClpA subunit of the Clp protease that functions in cellular protein quality control and can function in in numerous processes. ClpA mutants in some *Pseudomonas* species have cell envelope-related phenotypes (38, 39).

The HL mutants that secrete lipid into the medium (HLM01, HLM02, and HLM05) map to two loci. The gene inactivated in HLM05 (RSP1200) encodes an uncharacterized conserved protein that contains a CSP/antigen 5/Pr11 (CAP) domain. Homologues of RSP1200 are typically secreted, acting extracellularly in signal transduction or protein modification (40); some family members are known to bind lipids (41, 42). HLM01 and HLM02 contain disruptions in a sensor histidine kinase (NtrY) and its cognate response regulator (NtrX). The most recent phylogenetic analysis indicates that NtrX homologues are found across the bacterial phylogeny, with most homologues found in alpha-, beta-, gamma-, and deltaproteobacterial genomes (43). In systems where it has been studied, the NtrYX pathway has been implicated in controlling exopolysaccharide production (44), as well as regulating respiratory and anaerobically induced processes (45–49). Future analysis of *R. sphaeroides* NtrYX target genes can determine how they impinge on the cell envelope, lead to the HL phenotype, and result in extracellular production of fatty acids and possibly other membrane-associated compounds.

Finally, some HL mutants disrupt genes whose products cannot be linked to the cell envelope at this time. RSP3218 (inactivated in HLM03) encodes a nitroreductase that is predicted to function in vitamin B_12_ biosynthesis, RSP1422 (inactivated in HLM06) encodes a chromosome-partitioning protein, and RSP1056 (inactivated in HLM04) is a putative histidine kinase, but its cognate response regulator and cellular function are unknown. In these cases, additional experiments are needed to understand how these mutations lead to their phenotype. It is also possible that loss of the Tn*5*-mutated gene is not directly responsible for the observed HL phenotype. We have shown that loss of NrtYX leads to the HL phenotype observed in HLM01 and HLM02; however, other mutants may contain a secondary mutation causing or contributing to the observed phenotype.

### Lipid secretion by some HL mutants.

When *R. sphaeroides* naturally increases its lipid content, it sequesters the lipids in intracellular membranes, and so it was unexpected to find that several HL mutants secreted lipids. From analysis of other bacteria, lipid secretion often occurs by export of free fatty acids or outer membrane vesicle (OMV) formation (50–52). For the HL mutants with the highest levels of secreted lipid (HLM01, HLM02, and HLM05), lipid phosphorus assays indicated the presence of extracellular phospholipid (Fig. S5). The extracellular structures in HLM01 and HLM02 cultures (Fig. 3B, C, F, and G) do not resemble bacterial OMVs, which typically appear as 20- to 250-nm spherical vesicles (53). However, the medium of HLM05 does contain spherical vesicles in the 20- to 50-nm range (Fig. 3H). Further studies are needed to analyze the process of lipid secretion and the chemical composition of the secreted material.

### *R. sphaeroides* as an oleaginous bacterium.

The ability of *R. sphaeroides* to increase production of hydrophobic compounds has led to its use as a source of isoprenoids, quinones, and other chemicals (32, 54, 55). The utility of bacteria as microbial sources of valuable products is often enhanced by the ability to grow cells to high cell density. Growing HLM02 in a fed-batch bioreactor increased total fatty acid productivity ~8-fold over what is observed in batch culture. Additionally, in the fed-batch bioreactor, the total fatty acid content of HLM02 was ~33% of DCW, classifying this strain as an oleaginous bacterium. We are not aware of any previous examples of a microbe accumulating over 20% of its biomass as phospholipid; typically, oleaginous organisms accumulate triacylglycerols or wax esters (1, 56). The fatty acid yield for HLM02 in the fed-batch bioreactor was 24% of maximum theoretical yield, a substantial improvement over wild-type cells when one considers that this strain contains only one genetic lesion. Further increases in lipid productivity in this and other HL mutants may be possible with additional metabolic engineering, introducing other gene disruptions identified in this study (Table 1) or strategies that have been successful in other organisms to increase flux through fatty acid biosynthesis or decrease β-oxidation (22, 23).

Several of our observations may help increase the feasibility of lipid production, either in this or in other bacteria that can be engineered to contain cell envelope changes. First, bioproduct secretion can increase production beyond the amount that can fit within the cell and minimize any toxicity of the product. Second, bioproduct secretion could simplify harvesting, separation, and subsequent product processing (57, 58). In addition, at least one of the HL mutants (HLM02) retained its phenotype when grown on several different carbon sources, suggesting that its utility as a host will be retained when cells are grown on more complex media. Finally, we found that the HLM02 mutant could overproduce and secrete a novel furan-containing fatty acid that has potential value in the biofuel, biochemical, and pharmaceutical industries.

In sum, we have increased our knowledge of factors that control lipid production and created an oleaginous strain of *R. sphaeroides*, HLM02. We propose that in many of the HL mutants, alterations in the cell envelope lead to increased lipid content. The novel properties of these HL mutants also suggests that similar changes in the cell envelope could be used to increase production of lipids or hydrophobic products in other microbes.

## MATERIALS AND METHODS

### Bacterial strains and growth conditions.

*R. sphaeroides* strains (see Table S1 in the supplemental material) were grown in Sistrom’s minimal medium (SIS) (59), with 4 g/liter succinate or, where indicated, glucose, xylose, or lactate. Aerobic batch cultures of 10 to 20 ml were grown in 125-ml flasks with shaking at 200 rpm, at 30°C. For liquid photosynthetic growth (anaerobic), completely filled screw-cap tubes were incubated in front of an incandescent light box with a light intensity of 10 W/m^2^ measured through a red glass filter. Cultures were grown to an optical density at 600 nm (OD_600_) of 0.5 to 1.5 for analysis. *Escherichia coli* strains were grown at 37°C in LB broth (60). When necessary, media were supplemented with 50 µg/ml kanamycin, 25 µg/ml spectinomycin, 30 or 50 µg/ml trimethoprim (for *R. sphaeroides* and *E. coli*, respectively), and 10 µM isopropyl-β-d-1-thiogalactopyranoside (IPTG).

10.1128/mBio.00513-17.8 TABLE S1  Strains and plasmids used in this study. Download TABLE S1, PDF file, 0.04 MB. Copyright © 2017 Lemmer et al. 2017 Lemmer et al. This content is distributed under the terms of the Creative Commons Attribution 4.0 International license.

### Transposon mutagenesis and Nile red screening.

The transposon delivery plasmid pRL27 (61) was conjugated into *R. sphaeroides* Δ0382 using *E. coli* donor strain BW20767. Individual exconjugant colonies from SIS agar plates were inoculated into 200 µl SIS plus kanamycin in 96-well plates, grown in a humidified incubator with shaking at 30°C to saturation (3 days), and diluted into SIS containing 5µg/ml Nile red in Nunc 96-well black optical-bottom plates (Thermo Scientific). After ~14 h of incubation, fluorescence (excitation, 530 nm; emission, 580 nm) and absorbance (650 nm and 850 nm) were measured in an Infinite M1000 plate reader (Tecan). For strains with fluorescence divided by OD_650_ that was ≥60% higher than the plate average, Nile red staining was repeated with replicates before cellular fatty acid analysis (see "Analytical procedures" section below). Transposon insertion sites were identified by cloning transposon-containing fragments from BamHI-digested genomic DNA (61).

### Chemical sensitivity analysis.

Compounds (Table S2) were tested for their effects on growth of the parent strain to determine the highest doses that cause <30% growth reduction. Parent and HL mutant strains were grown in the presence of the chemicals, or dimethyl sulfoxide (DMSO) as a control, in 96-well plates at 30°C with shaking for 48 h. Final ODs were read at 595 nm on an Infinite F500 microplate reader (Tecan). For each strain, final ODs for each treatment were divided by the OD of the DMSO control for that strain to determine relative cell growth, and then the growth value for each treated culture was normalized by the parent strain growth under the same condition. Two-way clustering was performed with Cluster 3.0 and visualized with Java TreeView software (62).

10.1128/mBio.00513-17.9 TABLE S2  Compounds used for chemical sensitivity analysis. Download TABLE S2, XLSX file, 0.04 MB. Copyright © 2017 Lemmer et al. 2017 Lemmer et al. This content is distributed under the terms of the Creative Commons Attribution 4.0 International license.

10.1128/mBio.00513-17.10 TABLE S3  Relative fatty acid content of *R. sphaeroides* ΔChrRΔNtrYXpd. Download TABLE S3, PDF file, 0.02 MB. Copyright © 2017 Lemmer et al. 2017 Lemmer et al. This content is distributed under the terms of the Creative Commons Attribution 4.0 International license.

### Fed-batch bioreactor cultures.

Fed-batch cultures were grown in an Applikon biofermentor (3-liter autoclavable microbial BioBundle; Applikon Biotechnology) using an adapted SIS medium (ASIS). ASIS contained a 20-fold-higher concentration of xylose, a 25-fold-higher concentration of ammonium sulfate, a 2-fold-higher concentration of dipotassium phosphate, and 5-fold-higher concentrations of all other SIS components. For inoculation, 1 liter SIS was mixed with 50 ml of a succinate-grown batch culture. During operation, pH, DO, and temperature were monitored and controlled by external programmable logic controllers (ez-Control; Applikon Biotechnology). The pH was maintained between 6.95 and 7.05 with additions of 1 M H_2_SO_4_ or 10 M KOH, compressed air was used to provide aeration, temperature was maintained at 30°C, and ASIS medium was used to provide nutrients. DO was maintained below 5% of saturated air by fixed aeration rate and feeding ASIS.

### Analytical procedures.

As indicated, analysis was performed on either the whole culture or on the cells or medium after centrifugation (10,000 × *g* for 15 min at 4°C). Samples from the fed-batch reactor were diluted with deionized water before lipid extraction. Lipid extraction with chloroform-methanol, esterification, gas chromatography-mass spectrometry (GC-MS) analysis, and quantification were performed (63) using 2.5-ml samples. For lipid phosphorus measurements, dried lipid extracts from 2.5-ml samples were digested with perchloric acid before measuring phosphorus content (64). Organic acids and sugars were analyzed by high-performance liquid chromatography (HPLC) (65, 66). Samples were prepared by filtering aliquots of the culture with an 0.22-µm filter before injection into the HPLC. DCW was calculated by measuring chemical oxygen demand (COD) per liter and using the conversion factor of 1.47 g COD/g DCW, which was determined from the composition of *R. sphaeroides* 2.4.1 biomass (27), adjusted for the lack of PHB in the parent and HL mutant strains, C_5_H_9_._49_O_2_._23_N0._76_S0._01_P0._24_. COD was analyzed using high-range COD test kits (Hach) according to the manufacturer’s protocols. Medium fractions were stained with 5 µg/ml Nile red, and fluorescence was measured as described above for Nile red screening. LPS was measured using the Pierce *Limulus* amoebocyte lysate (LAL) chromogenic endotoxin quantitation kit (Thermo Scientific) according to the manufacturer’s protocol. To estimate LPS-associated fatty acid levels, the following conversion factors were used: 1 endotoxin unit (EU) of LPS equals 100 pg, 1 mol LPS equals 10,000 g, and *R. sphaeroides* LPS contains 5 acyl chains per molecule (67). *P* values for statistical significance were calculated by unpaired *t* test using GraphPad QuickCalcs.

### Microscopy.

For TEM whole mounts, 5 μl of cell suspension was applied to a TEM grid, poststained with a negative stain (NanoW; Nanoprobes), blotted after 30 s, and allowed to air dry. TEM samples were examined using a Tecnai T-12 TEM (FEI) operating at 120 kV with a LaB6 filament. Images were collected digitally with a 2x2K Ultrascan 1000 charge-coupled device (CCD) (Gatan).

For SIM analysis, cells were fixed by being added to an equal volume of 4% paraformaldehyde, incubated for 45 min, and washed twice with phosphate-buffered saline (PBS). For staining, 2.5 µl cell suspension, 42.5 µl PBS buffer, and 5 µl Nile red stock solution (1 mg/ml in ethanol) were mixed, incubated for 10 min, centrifuged, and resuspended in PBS. Samples were dropped onto polylysine-coated glass coverslips. Superresolution fluorescence images were collected with a Zeiss Elyra S1 structured illumination microscope. The 63× oil immersion objective, 488-nm wavelength laser fluorescence excitation source, and emission 495- to ~550-nm band-pass filter were used. Seventy-five or more cells per sample were measured by custom Matlab scripts.

### Strain construction.

Deletion of the *ntrX* and *ntrY* genes (RSP2839 and RSP2840) was used to create the ΔNtrYXpd strain using the nonreplicable integration vector pK18mobsacB (68). Both open reading frames (ORFs) plus ~1 kb of flanking DNA sequences on either side were amplified from genomic DNA with primers containing XbaI and HindIII restriction sites. This PCR product was inserted into pK18mobsacB to create plasmid pKCL20. The entire coding regions of RSP2839 and RSP2840 were deleted from the plasmid by performing PCR with primers facing outward from the upstream end of RSP2839 and the downstream end of RSP2840 and ligation of the resulting fragment with T4 DNA ligase (Promega) to create pKCL21. *E. coli* S17-1 was used for conjugation of pKCL21 into *R. sphaeroides* Δ0382 (20). Single crossovers were selected by kanamycin resistance, and double crossovers were selected by loss of sucrose sensitivity. The ΔChrRΔNtrYXpd strain was created by deleting *chrR* using plasmid pJDN27, as described previously (69).

## References

[1] Liang MH, Jiang JG 2013 Advancing oleaginous microorganisms to produce lipid via metabolic engineering technology. Prog Lipid Res 52:395–408. doi:10.1016/j.plipres.2013.05.002. 23685199

[2] Sawangkeaw R, Ngamprasertsith S 2013 A review of lipid-based biomasses as feedstocks for biofuels production. Renew Sustain Energy Rev 25:97–108. doi:10.1016/j.rser.2013.04.007.

[3] Zhang YM, Rock CO 2010 A rainbow coalition of lipid transcriptional regulators. Mol Microbiol 78:5–8. doi:10.1111/j.1365-2958.2010.07349.x. 20941840 PMC2967205

[4] Levering J, Broddrick J, Zengler K 2015 Engineering of oleaginous organisms for lipid production. Curr Opin Biotechnol 36:32–39. doi:10.1016/j.copbio.2015.08.001. 26319892

[5] Kosa M, Ragauskas AJ 2011 Lipids from heterotrophic microbes: advances in metabolism research. Trends Biotechnol 29:53–61. doi:10.1016/j.tibtech.2010.11.002. 21146236

[6] Blatti JL, Michaud J, Burkart MD 2013 Engineering fatty acid biosynthesis in microalgae for sustainable biodiesel. Curr Opin Chem Biol 17:496–505. doi:10.1016/j.cbpa.2013.04.007. 23683348

[7] d’Espaux L, Mendez-Perez D, Li R, Keasling JD 2015 Synthetic biology for microbial production of lipid-based biofuels. Curr Opin Chem Biol 29:58–65. doi:10.1016/j.cbpa.2015.09.009. 26479184

[8] Steen EJ, Kang Y, Bokinsky G, Hu Z, Schirmer A, McClure A, Del Cardayre SB, Keasling JD 2010 Microbial production of fatty-acid-derived fuels and chemicals from plant biomass. Nature 463:559–562. doi:10.1038/nature08721. 20111002

[9] Schirmer A, Rude MA, Li X, Popova E, del Cardayre SB 2010 Microbial biosynthesis of alkanes. Science 329:559–562. doi:10.1126/science.1187936. 20671186

[10] Goh EB, Baidoo EE, Keasling JD, Beller HR 2012 Engineering of bacterial methyl ketone synthesis for biofuels. Appl Environ Microbiol 78:70–80. doi:10.1128/AEM.06785-11. 22038610 PMC3255637

[11] Kalscheuer R, Stölting T, Steinbüchel A 2006 Microdiesel: *Escherichia coli* engineered for fuel production. Microbiology 152:2529–2536. doi:10.1099/mic.0.29028-0. 16946248

[12] Choi YJ, Lee SY 2013 Microbial production of short-chain alkanes. Nature 502:571–574. doi:10.1038/nature12536. 24077097

[13] Feng X, Lian J, Zhao H 2015 Metabolic engineering of *Saccharomyces cerevisiae* to improve 1-hexadecanol production. Metab Eng 27:10–19. doi:10.1016/j.ymben.2014.10.001. 25466225

[14] Chory J, Donohue TJ, Varga AR, Staehelin LA, Kaplan S 1984 Induction of the photosynthetic membranes of *Rhodopseudomonas sphaeroides*: biochemical and morphological studies. J Bacteriol 159:540–554. 6611335 10.1128/jb.159.2.540-554.1984 PMC215678

[15] Tavano CL, Donohue TJ 2006 Development of the bacterial photosynthetic apparatus. Curr Opin Microbiol 9:625–631. doi:10.1016/j.mib.2006.10.005. 17055774 PMC2765710

[16] Kiley PJ, Kaplan S 1988 Molecular genetics of photosynthetic membrane biosynthesis in *Rhodobacter sphaeroides*. Microbiol Rev 52:50–69. 3280966 10.1128/mr.52.1.50-69.1988 PMC372705

[17] Lemmer KC, Dohnalkova AC, Noguera DR, Donohue TJ 2015 Oxygen-dependent regulation of bacterial lipid production. J Bacteriol 197:1649–1658. doi:10.1128/JB.02510-14. 25733615 PMC4403652

[18] Zeilstra-Ryalls J, Gomelsky M, Eraso JM, Yeliseev A, O’Gara J, Kaplan S 1998 Control of photosystem formation in *Rhodobacter sphaeroides*. J Bacteriol 180:2801–2809. 9603864 10.1128/jb.180.11.2801-2809.1998 PMC107241

[19] Zeilstra-Ryalls JH, Gomelsky M, Yeliseev AA, Eraso JM, Kaplan S 1998 Transcriptional regulation of photosynthesis operons in *Rhodobacter sphaeroides* 2.4.1. Methods Enzymol 297:151–166. 9750207 10.1016/s0076-6879(98)97012-4

[20] Yilmaz LS, Kontur WS, Sanders AP, Sohmen U, Donohue TJ, Noguera DR 2010 Electron partitioning during light- and nutrient-powered hydrogen production by *Rhodobacter sphaeroides*. Bioenerg Res 3:55–66. doi:10.1007/s12155-009-9072-8.

[21] Rott MA, Witthuhn VC, Schilke BA, Soranno M, Ali A, Donohue TJ 1993 Genetic evidence for the role of isocytochrome *c*_2_ in photosynthetic growth of *Rhodobacter sphaeroides* Spd mutants. J Bacteriol 175:358–366. doi:10.1128/jb.175.2.358-366.1993. 8380401 PMC196149

[22] Janssen HJ, Steinbüchel A 2014 Fatty acid synthesis in *Escherichia coli* and its applications towards the production of fatty acid based biofuels. Biotechnol Biofuels 7:7. doi:10.1186/1754-6834-7-7. 24405789 PMC3896788

[23] Lennen RM, Pfleger BF 2012 Engineering *Escherichia coli* to synthesize free fatty acids. Trends Biotechnol 30:659–667. doi:10.1016/j.tibtech.2012.09.006. 23102412 PMC3856887

[24] Andrusiak K, Piotrowski JS, Boone C 2012 Chemical-genomic profiling: systematic analysis of the cellular targets of bioactive molecules. Bioorg Med Chem 20:1952–1960. doi:10.1016/j.bmc.2011.12.023. 22261022

[25] Vaara M 1993 Outer membrane permeability barrier to azithromycin, clarithromycin, and roxithromycin in gram-negative enteric bacteria. Antimicrob Agents Chemother 37:354–356. doi:10.1128/AAC.37.2.354. 8383945 PMC187668

[26] Zhang L, Song J, Cavigiolio G, Ishida BY, Zhang S, Kane JP, Weisgraber KH, Oda MN, Rye KA, Pownall HJ, Ren G 2011 Morphology and structure of lipoproteins revealed by an optimized negative-staining protocol of electron microscopy. J Lipid Res 52:175–184. doi:10.1194/jlr.D010959. 20978167 PMC2999936

[27] Imam S, Yilmaz S, Sohmen U, Gorzalski AS, Reed JL, Noguera DR, Donohue TJ 2011 iRsp1095: a genome-scale reconstruction of the *Rhodobacter sphaeroides* metabolic network. BMC Syst Biol 5:116. doi:10.1186/1752-0509-5-116. 21777427 PMC3152904

[28] Agler MT, Wrenn BA, Zinder SH, Angenent LT 2011 Waste to bioproduct conversion with undefined mixed cultures: the carboxylate platform. Trends Biotechnol 29:70–78. doi:10.1016/j.tibtech.2010.11.006. 21190748

[29] Lau MW, Gunawan C, Dale BE 2009 The impacts of pretreatment on the fermentability of pretreated lignocellulosic biomass: a comparative evaluation between ammonia fiber expansion and dilute acid pretreatment. Biotechnol Biofuels 2:30–30. doi:10.1186/1754-6834-2-30. 19961578 PMC2799388

[30] Lemke RA, Peterson AC, Ziegelhoffer EC, Westphall MS, Tjellström H, Coon JJ, Donohue TJ 2014 Synthesis and scavenging role of furan fatty acids. Proc Natl Acad Sci U S A 111:E3450–E3457. doi:10.1073/pnas.1405520111. PMC4143029 25092314

[31] Shiloach J, Fass R 2005 Growing *E. coli* to high cell density—a historical perspective on method development. Biotechnol Adv 23:345–357. doi:10.1016/j.biotechadv.2005.04.004. 15899573

[32] Yen HW, Feng CY, Kang JL 2010 Cultivation of *Rhodobacter sphaeroides* in the stirred bioreactor with different feeding strategies for CoQ_10_ production. Appl Biochem Biotechnol 160:1441–1449. doi:10.1007/s12010-009-8576-1. 19277486

[33] Zeiger L, Grammel H 2010 Model-based high cell density cultivation of *Rhodospirillum rubrum* under respiratory dark conditions. Biotechnol Bioeng 105:729–739. doi:10.1002/bit.22589. 19882736

[34] Seo DJ, Chung BH, Hwang YD, Park YH 1992 Glucose-limited fed-batch culture of *Escherichia coli* for production of recombinant human interleukin-2 with the DO-stat method. J Ferment Bioeng 74:196–198. doi:10.1016/0922-338X(92)90085-9.

[35] Lyu ZX, Zhao XS 2015 Periplasmic quality control in biogenesis of outer membrane proteins. Biochem Soc Trans 43:133–138. doi:10.1042/BST20140217. 25849907

[36] Bateman A, Bycroft M 2000 The structure of a LysM domain from *E. coli* membrane-bound lytic murein transglycosylase D (MltD). J Mol Biol 299:1113–1119. doi:10.1006/jmbi.2000.3778. 10843862

[37] Sperisen P, Schmid CD, Bucher P, Zilian O 2005 Stealth proteins: in silico identification of a novel protein family rendering bacterial pathogens invisible to host immune defense. PLoS Comput Biol 1:e63. doi:10.1371/journal.pcbi.0010063. 16299590 PMC1285062

[38] Song C, Sundqvist G, Malm E, de Bruijn I, Kumar A, van de Mortel J, Bulone V, Raaijmakers JM 2015 Lipopeptide biosynthesis in *Pseudomonas fluorescens* is regulated by the protease complex ClpAP. BMC Microbiol 15:29. doi:10.1186/s12866-015-0367-y. 25885431 PMC4332742

[39] Goff M, Nikodinovic-Runic J, O’Connor KE 2009 Characterization of temperature-sensitive and lipopolysaccharide overproducing transposon mutants of *Pseudomonas putida* CA-3 affected in PHA accumulation. FEMS Microbiol Lett 292:297–305. doi:10.1111/j.1574-6968.2009.01504.x. 19187205

[40] Gibbs GM, Roelants K, O’Bryan MK 2008 The CAP superfamily: cysteine-rich secretory proteins, antigen 5, and pathogenesis-related 1 proteins—roles in reproduction, cancer, and immune defense. Endocr Rev 29:865–897. doi:10.1210/er.2008-0032. 18824526

[41] Choudhary V, Schneiter R 2012 Pathogen-related yeast (PRY) proteins and members of the CAP superfamily are secreted sterol-binding proteins. Proc Natl Acad Sci U S A 109:16882–16887. doi:10.1073/pnas.1209086109. 23027975 PMC3479496

[42] Van Galen J, Van Balkom BW, Serrano RL, Kaloyanova D, Eerland R, Stüven E, Helms JB 2010 Binding of GAPR-1 to negatively charged phospholipid membranes: unusual binding characteristics to phosphatidylinositol. Mol Membr Biol 27:81–91. doi:10.3109/09687680903507080. 20095951

[43] Atack JM, Srikhanta YN, Djoko KY, Welch JP, Hasri NH, Steichen CT, Vanden Hoven RN, Grimmond SM, Othman DS, Kappler U, Apicella MA, Jennings MP, Edwards JL, McEwan AG 2013 Characterization of an *ntrX* mutant of *Neisseria gonorrhoeae* reveals a response regulator that controls expression of respiratory enzymes in oxidase-positive proteobacteria. J Bacteriol 195:2632–2641. doi:10.1128/JB.02062-12. 23564168 PMC3676050

[44] Wang D, Xue H, Wang Y, Yin R, Xie F, Luo L 2013 The *Sinorhizobium meliloti* ntrX gene is involved in succinoglycan production, motility, and symbiotic nodulation on alfalfa. Appl Environ Microbiol 79:7150–7159. doi:10.1128/AEM.02225-13. 24038694 PMC3837732

[45] Ishida ML, Assumpção MC, Machado HB, Benelli EM, Souza EM, Pedrosa FO 2002 Identification and characterization of the two-component NtrY/NtrX regulatory system in *Azospirillum brasilense*. Braz J Med Biol Res 35:651–661. doi:10.1590/S0100-879X2002000600004. 12045829

[46] Pawlowski K, Klosse U, de Bruijn FJ 1991 Characterization of a novel *Azorhizobium caulinodans* ORS571 two-component regulatory system, NtrY/NtrX, involved in nitrogen fixation and metabolism. Mol Gen Genet 231:124–138. doi:10.1007/BF00293830. 1661370

[47] Carrica MDC, Fernandez I, Martí MA, Paris G, Goldbaum FA 2012 The NtrY/X two-component system of *Brucella* spp. acts as a redox sensor and regulates the expression of nitrogen respiration enzymes. Mol Microbiol 85:39–50. doi:10.1111/j.1365-2958.2012.08095.x. 22582926

[48] Carrica MDC, Fernandez I, Sieira R, Paris G, Goldbaum FA 2013 The two-component systems PrrBA and NtrYX co-ordinately regulate the adaptation of *Brucella abortus* to an oxygen-limited environment. Mol Microbiol 88:222–233. doi:10.1111/mmi.12181. 23527685

[49] Gregor J, Zeller T, Balzer A, Haberzettl K, Klug G 2007 Bacterial regulatory networks include direct contact of response regulator proteins: interaction of RegA and NtrX in *Rhodobacter capsulatus*. J Mol Microbiol Biotechnol 13:126–139. doi:10.1159/000103604. 17693720

[50] Kulp A, Kuehn MJ 2010 Biological functions and biogenesis of secreted bacterial outer membrane vesicles. Annu Rev Microbiol 64:163–184. doi:10.1146/annurev.micro.091208.073413. 20825345 PMC3525469

[51] Lennen RM, Braden DJ, West RA, Dumesic JA, Pfleger BF 2010 A process for microbial hydrocarbon synthesis: overproduction of fatty acids in *Escherichia coli* and catalytic conversion to alkanes. Biotechnol Bioeng 106:193–202. doi:10.1002/bit.22660. 20073090 PMC3833807

[52] Ledesma-Amaro R, Dulermo R, Niehus X, Nicaud JM 2016 Combining metabolic engineering and process optimization to improve production and secretion of fatty acids. Metab Eng 38:38–46. doi:10.1016/j.ymben.2016.06.004. 27301328

[53] Schwechheimer C, Kuehn MJ 2015 Outer-membrane vesicles from Gram-negative bacteria: biogenesis and functions. Nat Rev Microbiol 13:605–619. doi:10.1038/nrmicro3525. 26373371 PMC5308417

[54] Kien NB, Kong IS, Lee MG, Kim JK 2010 Coenzyme Q10 production in a 150-l reactor by a mutant strain of *Rhodobacter sphaeroides*. J Ind Microbiol Biotechnol 37:521–529. doi:10.1007/s10295-010-0699-4. 20195885

[55] Sangkharak K, Prasertsan P 2007 Optimization of polyhydroxybutyrate production from a wild type and two mutant strains of *Rhodobacter sphaeroides* using statistical method. J Biotechnol 132:331–340. doi:10.1016/j.jbiotec.2007.07.721. 17765994

[56] Wältermann M, Hinz A, Robenek H, Troyer D, Reichelt R, Malkus U, Galla HJ, Kalscheuer R, Stöveken T, von Landenberg P, Steinbüchel A 2005 Mechanism of lipid-body formation in prokaryotes: how bacteria fatten up. Mol Microbiol 55:750–763. doi:10.1111/j.1365-2958.2004.04441.x. 15661001

[57] Caspeta L, Nielsen J 2013 Economic and environmental impacts of microbial biodiesel. Nat Biotechnol 31:789–793. doi:10.1038/nbt.2683. 24022152

[58] Arora R 2012 Microbial biotechnology: energy and environment. Cambridge CAB International, Wallingford, Oxfordshire, United Kingdom.

[59] Sistrom WR 1960 A requirement for sodium in the growth of *Rhodopseudomonas spheroides*. J Gen Microbiol 22:778–785. doi:10.1099/00221287-22-3-778. 14447230

[60] Miller JH 1992, A short course in bacterial genetics: a laboratory manual and handbook for *Escherichia coli* and related bacteria. Cold Spring Harbor Laboratory Press, Plainview, NY.

[61] Larsen RA, Wilson MM, Guss AM, Metcalf WW 2002 Genetic analysis of pigment biosynthesis in *Xanthobacter autotrophicus* Py2 using a new, highly efficient transposon mutagenesis system that is functional in a wide variety of bacteria. Arch Microbiol 178:193–201. doi:10.1007/s00203-002-0442-2. 12189420

[62] Saldanha AJ 2004 Java TreeView—extensible visualization of microarray data. Bioinformatics 20:3246–3248. doi:10.1093/bioinformatics/bth349. 15180930

[63] Lennon CW, Lemmer KC, Irons JL, Sellman MI, Donohue TJ, Gourse RL, Ross W 2014 A *Rhodobacter sphaeroides* protein mechanistically similar to *Escherichia coli* DksA regulates photosynthetic growth. mBio 5:e01105-14. doi:10.1128/mBio.01105-14. 24781745 PMC4010833

[64] Rouser G, Fkeischer S, Yamamoto A 1970 Two dimensional thin layer chromatographic separation of polar lipids and determination of phospholipids by phosphorus analysis of spots. Lipids 5:494–496. doi:10.1007/BF02531316. 5483450

[65] Austin S, Kontur WS, Ulbrich A, Oshlag JZ, Zhang W, Higbee A, Zhang Y, Coon JJ, Hodge DB, Donohue TJ, Noguera DR 2015 Metabolism of multiple aromatic compounds in corn stover hydrolysate by *Rhodopseudomonas palustris*. Environ Sci Technol 49:8914–8922. doi:10.1021/acs.est.5b02062. 26121369 PMC5031247

[66] Schwalbach MS, Keating DH, Tremaine M, Marner WD, Zhang Y, Bothfeld W, Higbee A, Grass JA, Cotten C, Reed JL, da Costa Sousa L, Jin M, Balan V, Ellinger J, Dale B, Kiley PJ, Landick R 2012 Complex physiology and compound stress responses during fermentation of alkali-pretreated corn stover hydrolysate by an *Escherichia coli* ethanologen. Appl Environ Microbiol 78:3442–3457. doi:10.1128/AEM.07329-11. 22389370 PMC3346445

[67] Kaltashov IA, Doroshenko V, Cotter RJ, Takayama K, Qureshi N 1997 Confirmation of the structure of lipid A derived from the lipopolysaccharide of *Rhodobacter sphaeroides* by a combination of MALDI, LSIMS, and tandem mass spectrometry. Anal Chem 69:2317–2322. doi:10.1021/ac9612943. 9212704

[68] Schäfer A, Tauch A, Jäger W, Kalinowski J, Thierbach G, Pühler A 1994 Small mobilizable multi-purpose cloning vectors derived from the *Escherichia coli* plasmids pK18 and pK19: selection of defined deletions in the chromosome of *Corynebacterium glutamicum*. Gene 145:69–73. doi:10.1016/0378-1119(94)90324-7. 8045426

[69] Newman JD, Falkowski MJ, Schilke BA, Anthony LC, Donohue TJ 1999 The *Rhodobacter sphaeroides* ECF sigma factor, σ^E^, and the target promoters *cycA* P3 and *rpoE* P1. J Mol Biol 294:307–320. doi:10.1006/jmbi.1999.3263. 10610760

