# Supplementary figures and images for: Mutations That Alter the Bacterial Cell Envelope Increase Lipid Production
Source: mBio. 2017 May 23;8(3):e00513-17. doi: 10.1128/mBio.00513-17 (PMC5442454; doi:10.1128/mBio.00513-17)

**A****neomycin**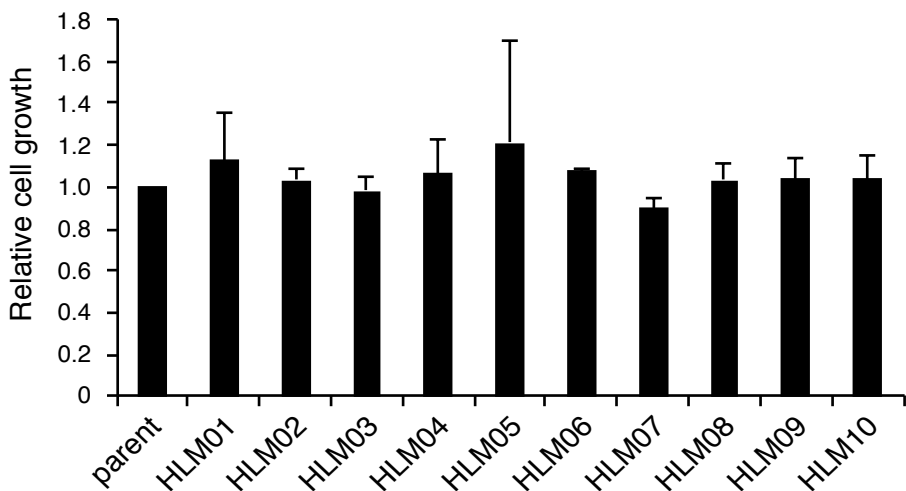**B****SDS**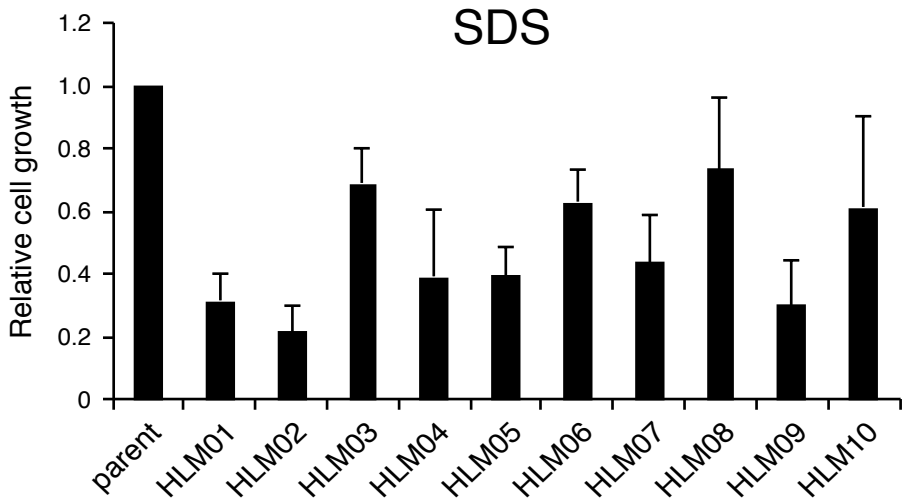**C****amoxicillin**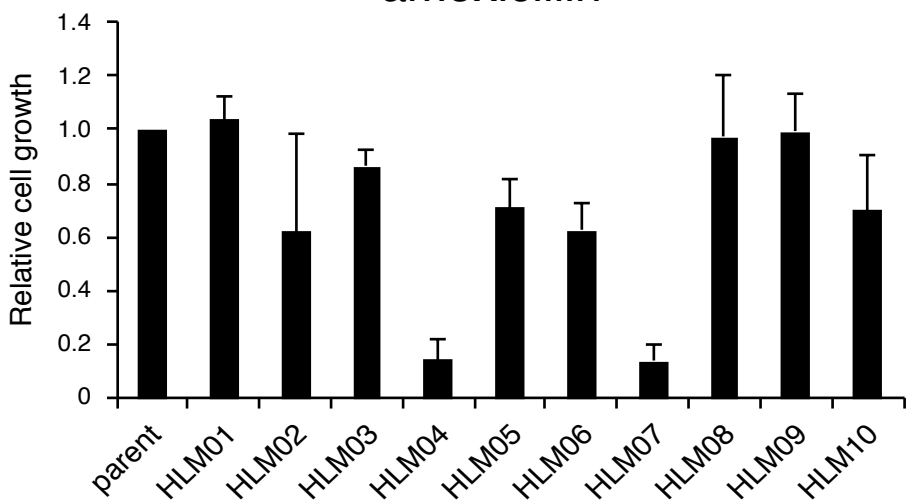

Supplement: FIG S1 [file mbo003173316sf1.pdf]

HLM08

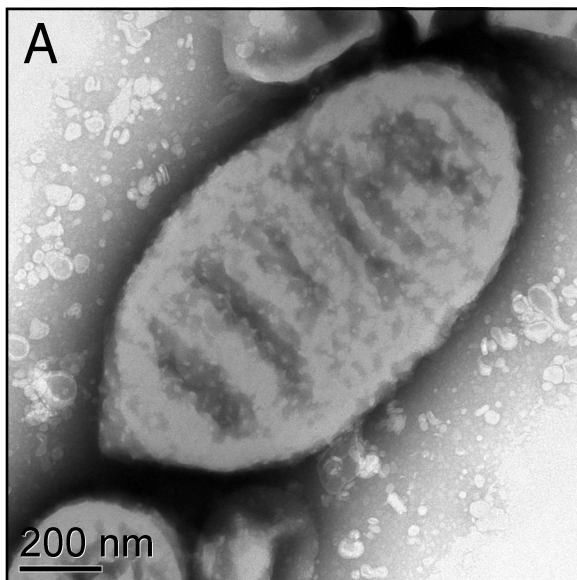

HLM08

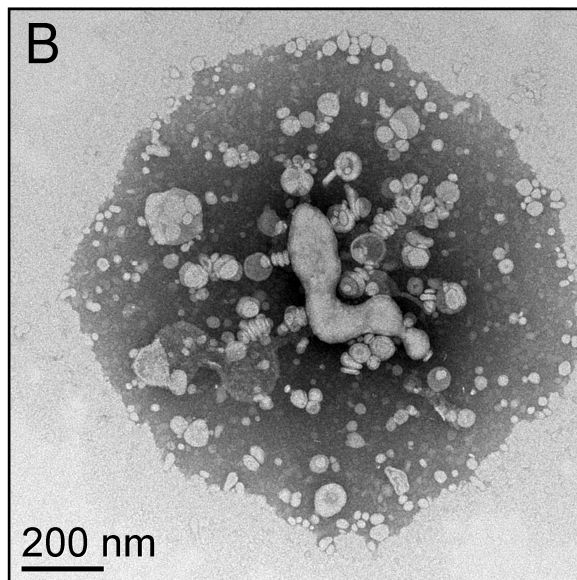

HLM03

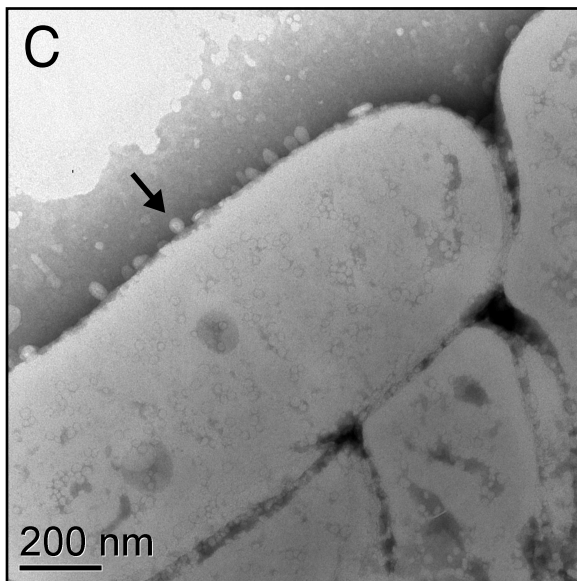

HLM09

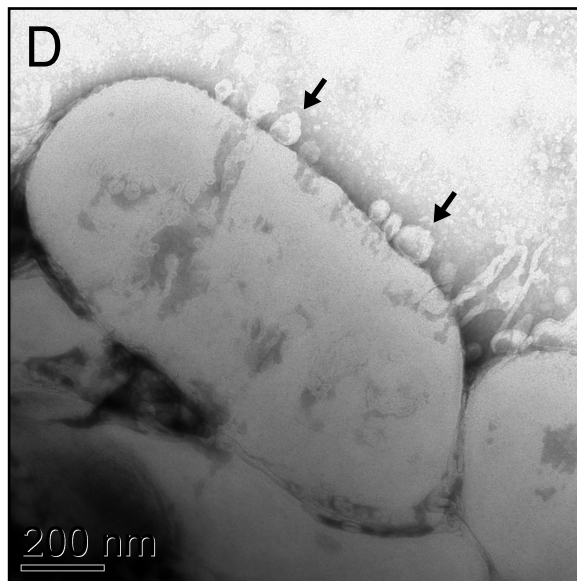

Supplement: FIG S2 [file mbo003173316sf2.pdf]

parent

HLM04

HLM07

TEM

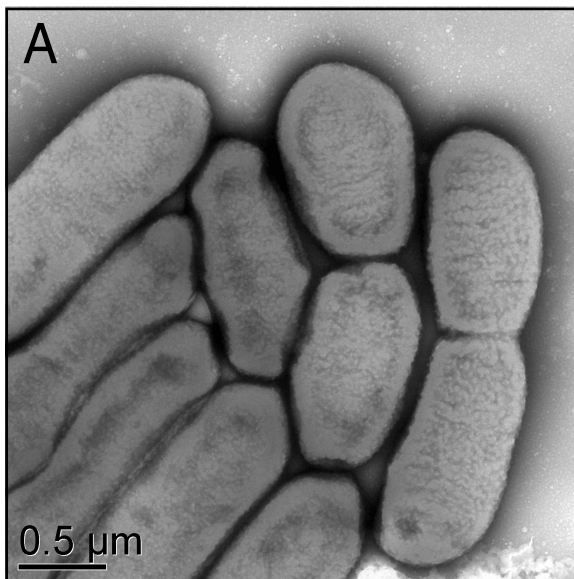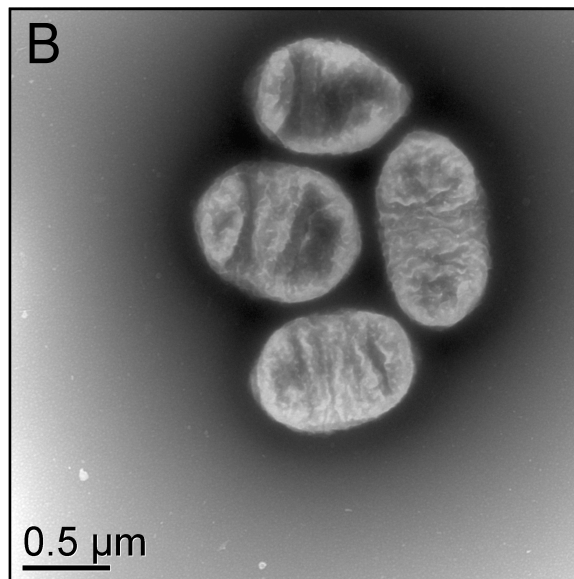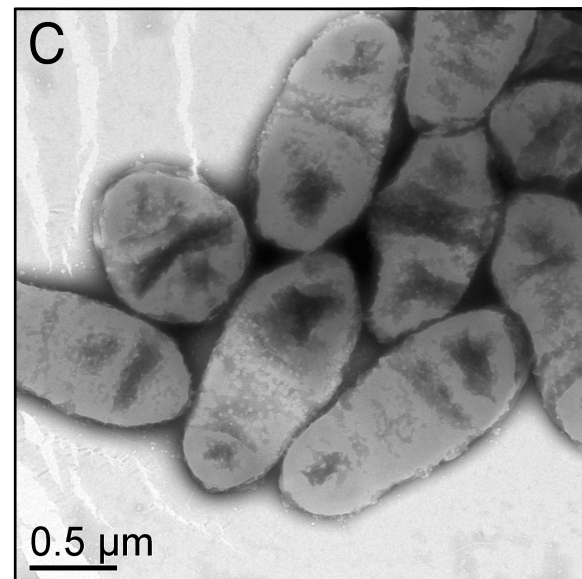

SIM

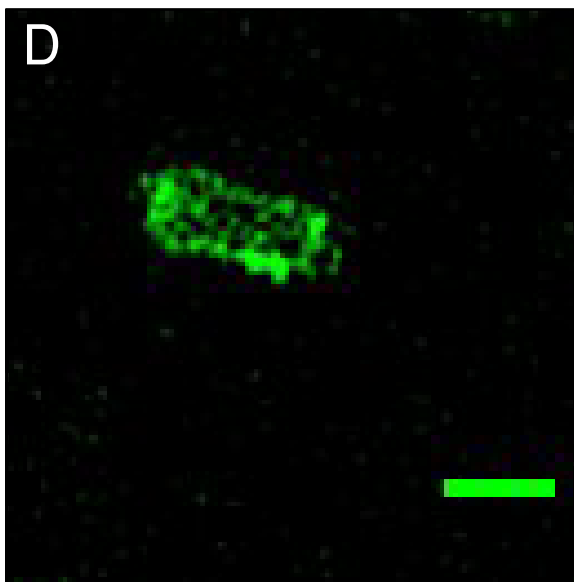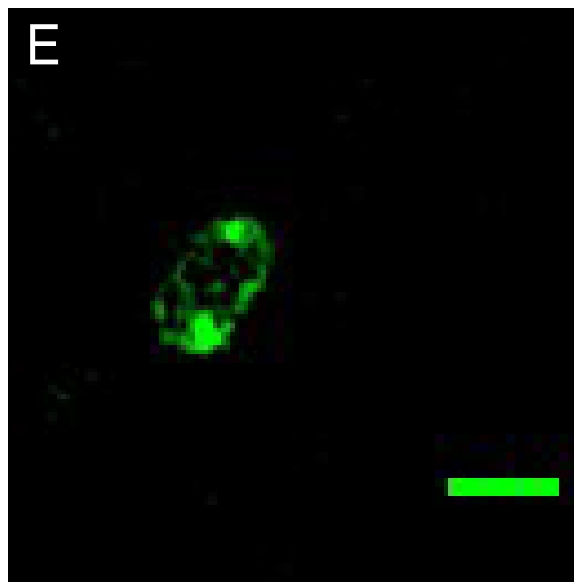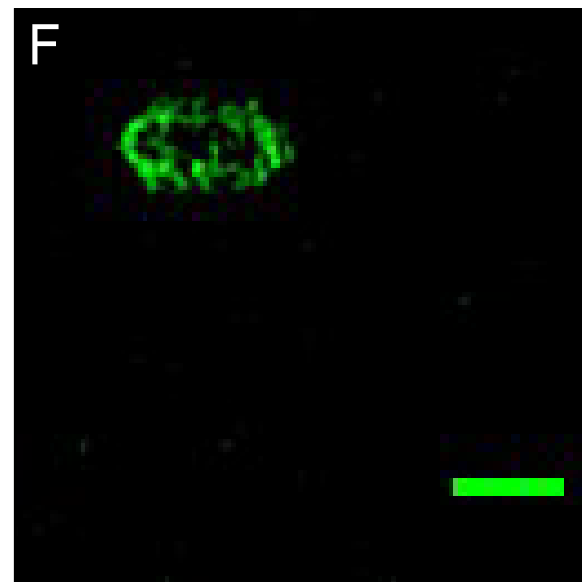

Supplement: FIG S3 [file mbo003173316sf3.pdf]

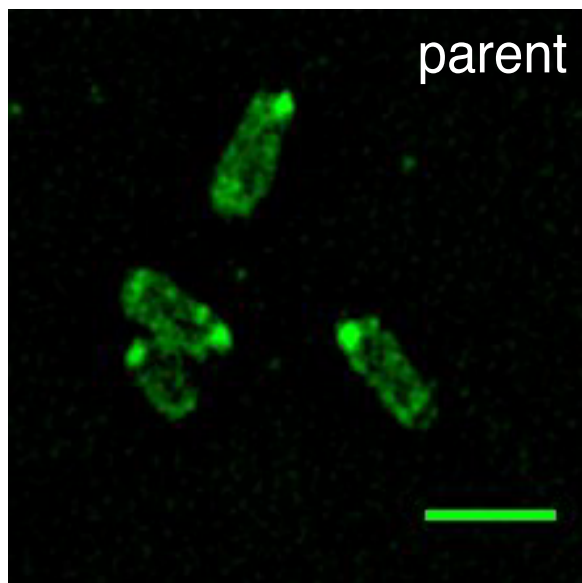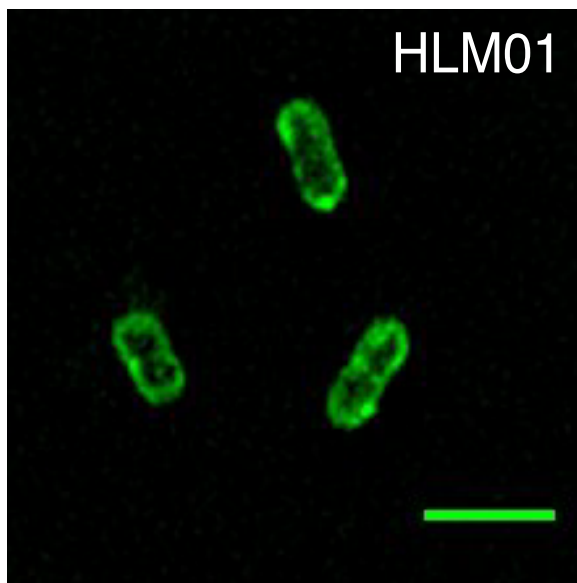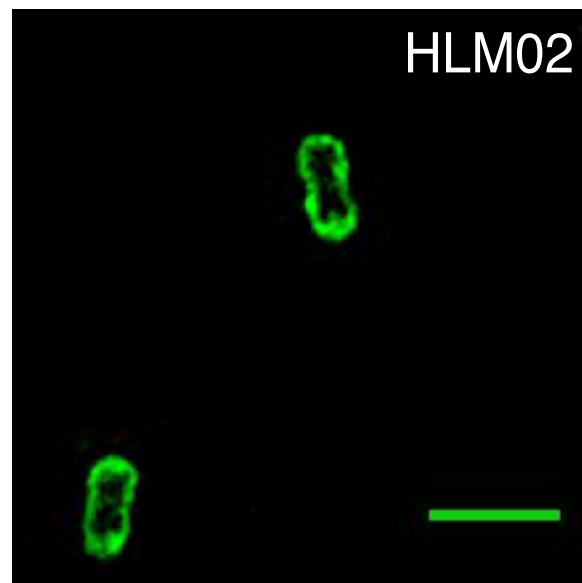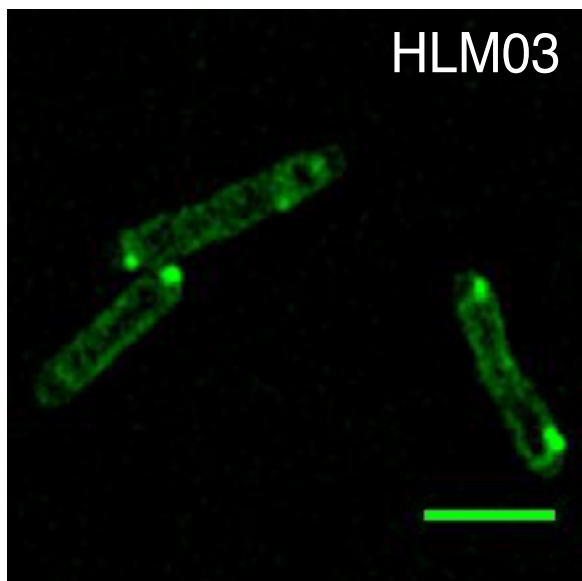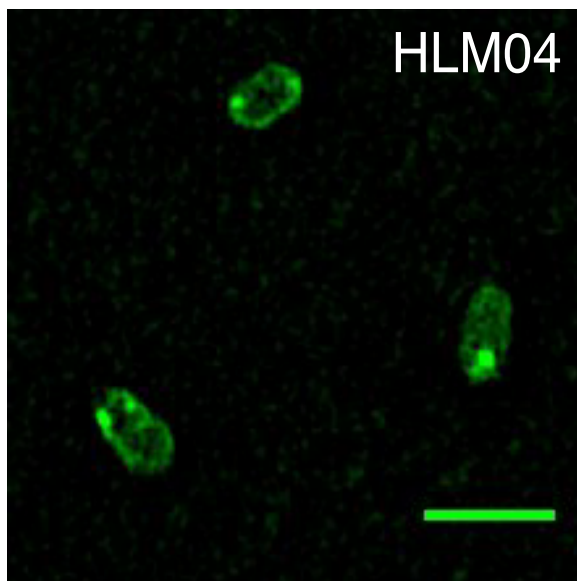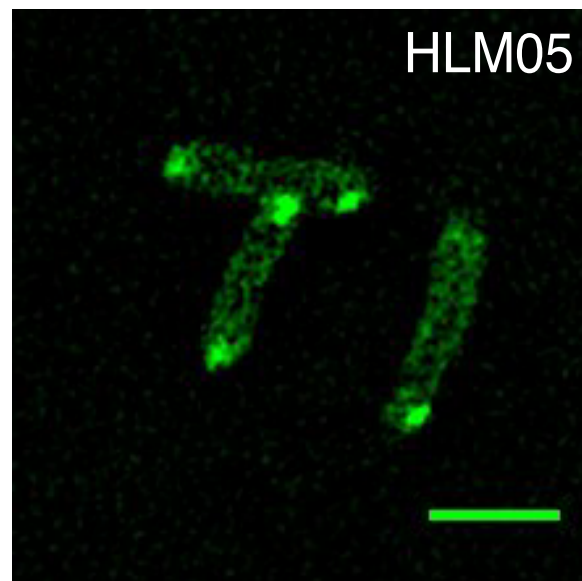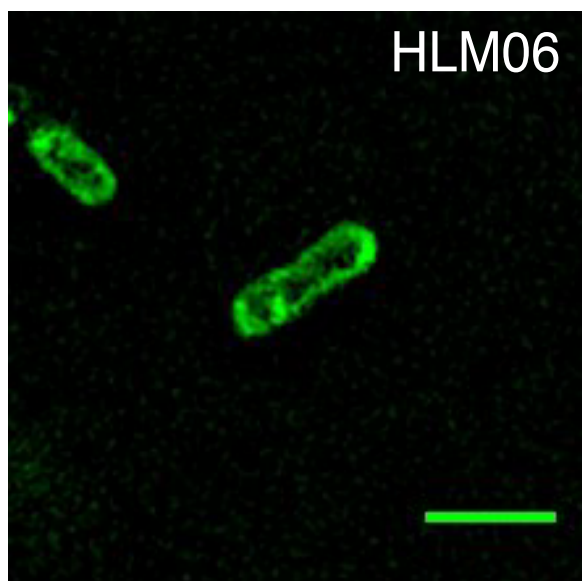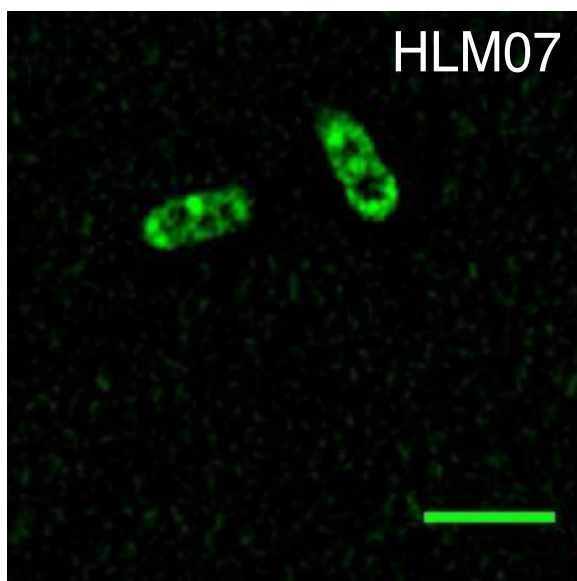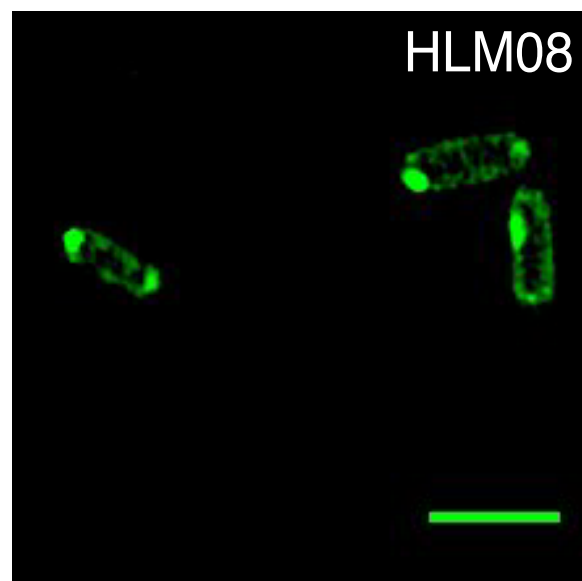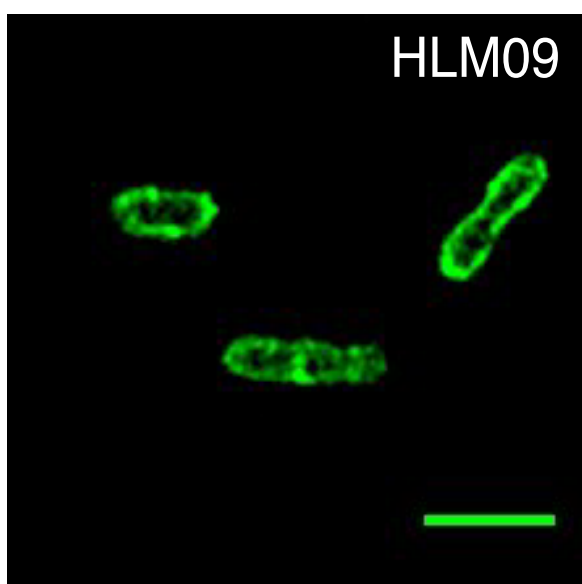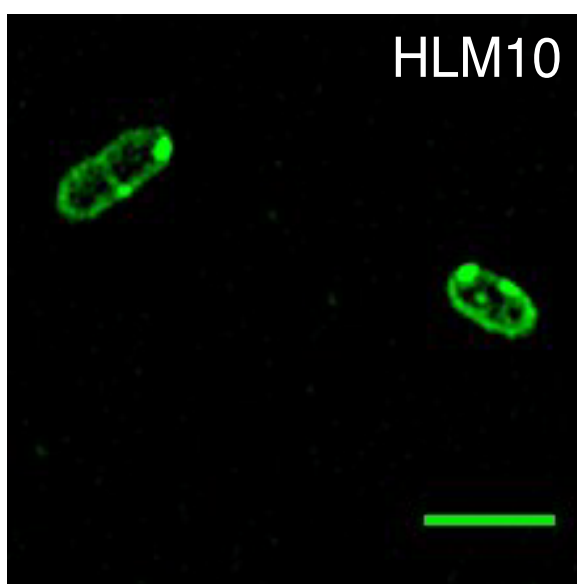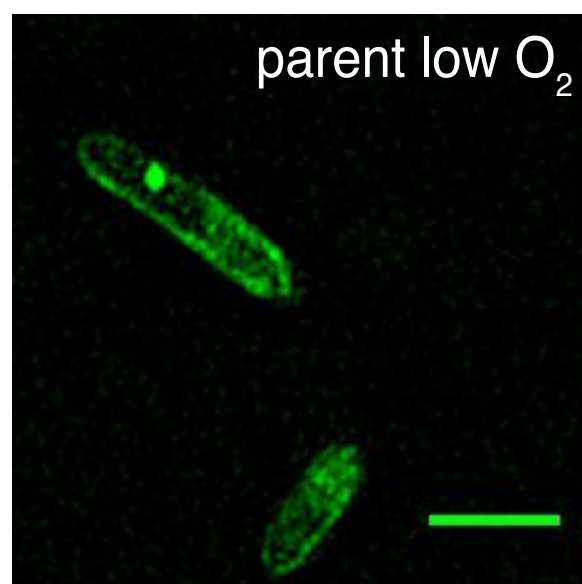

Supplement: FIG S4 [file mbo003173316sf4.pdf]

A

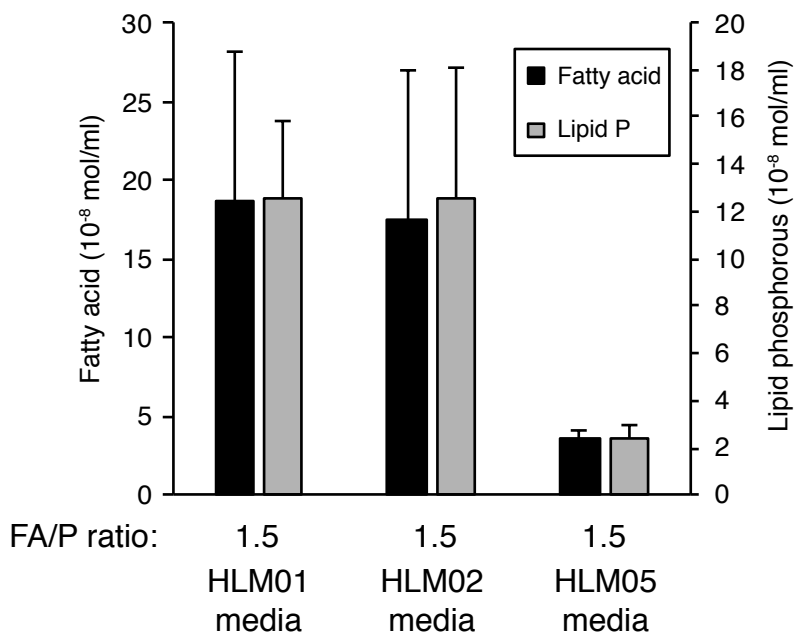

B

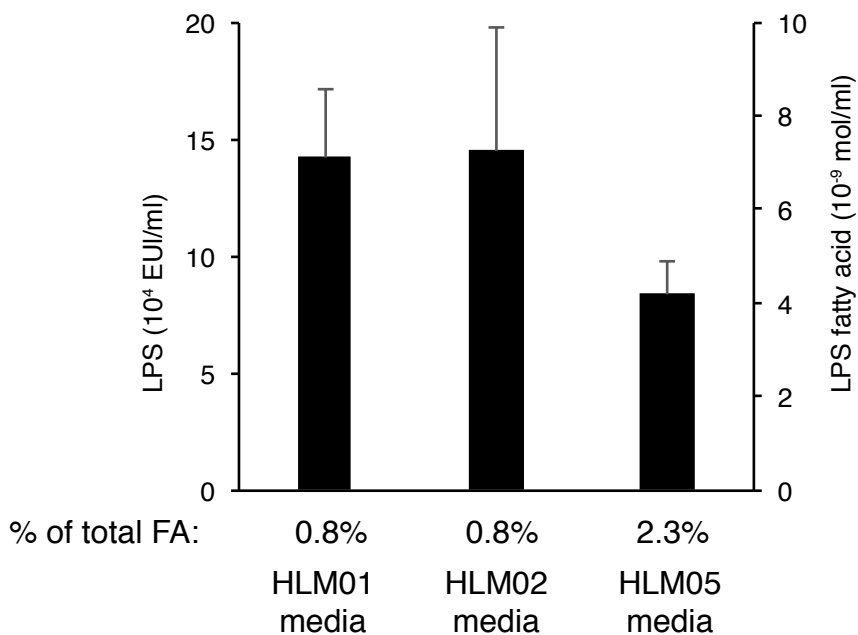

Supplement: FIG S5 [file mbo003173316sf5.pdf]

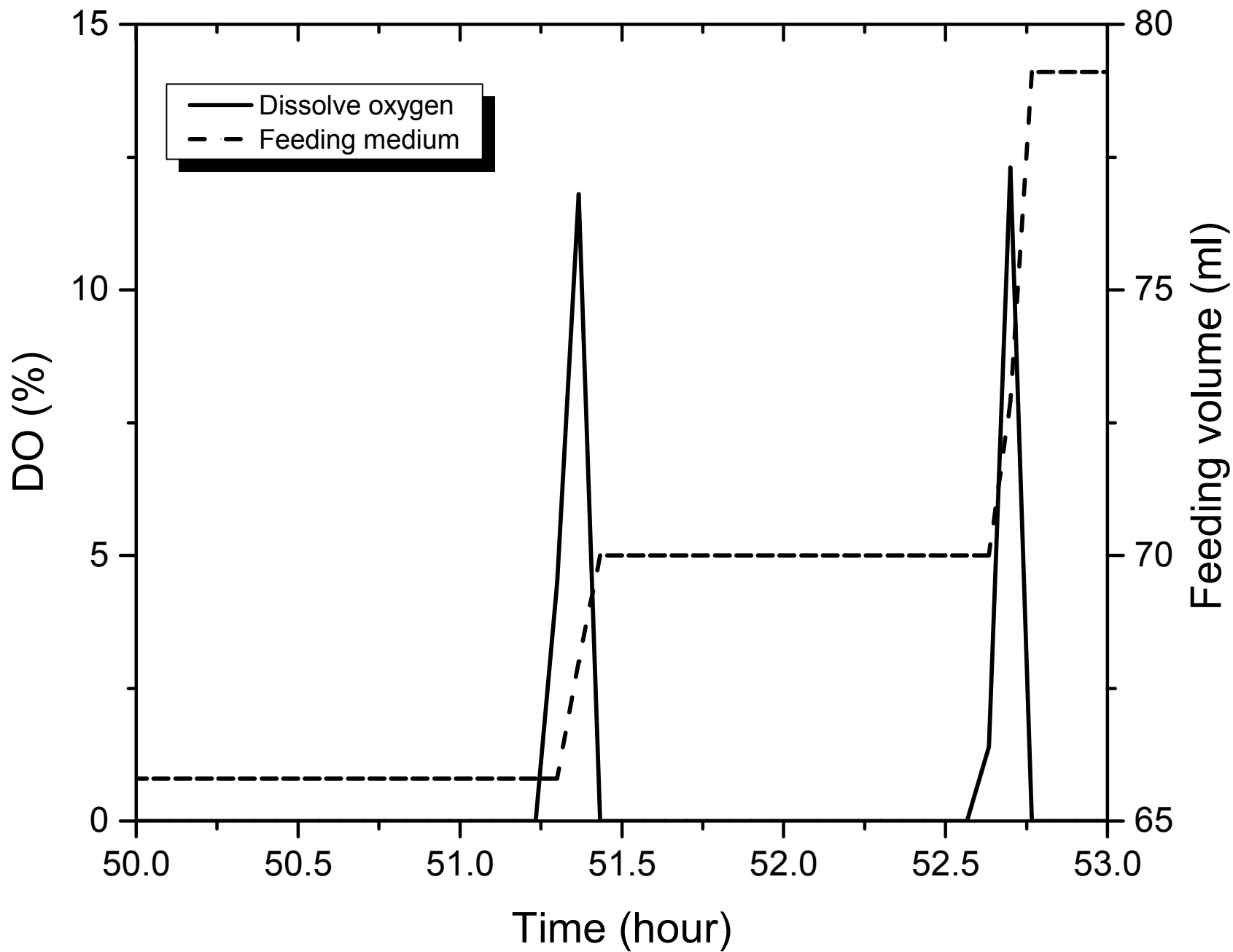

Supplement: FIG S6 [file mbo003173316sf6.pdf]

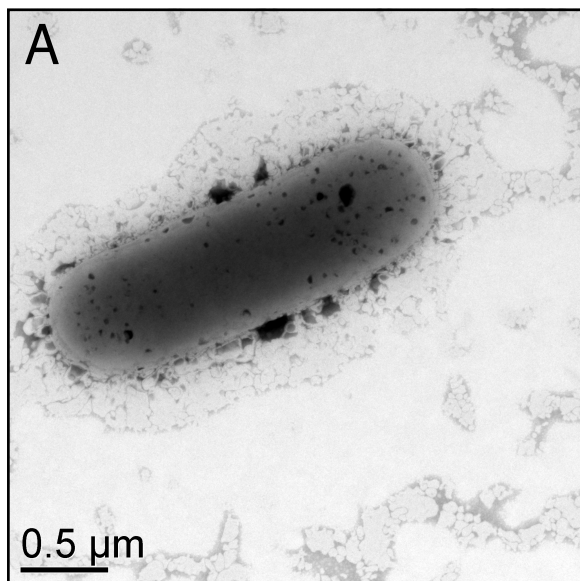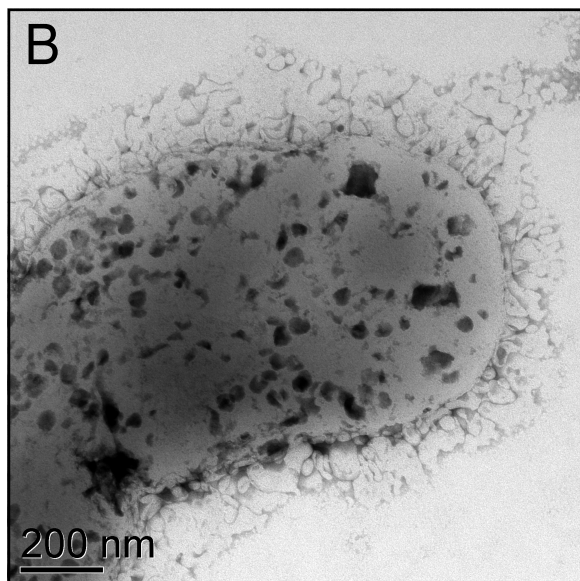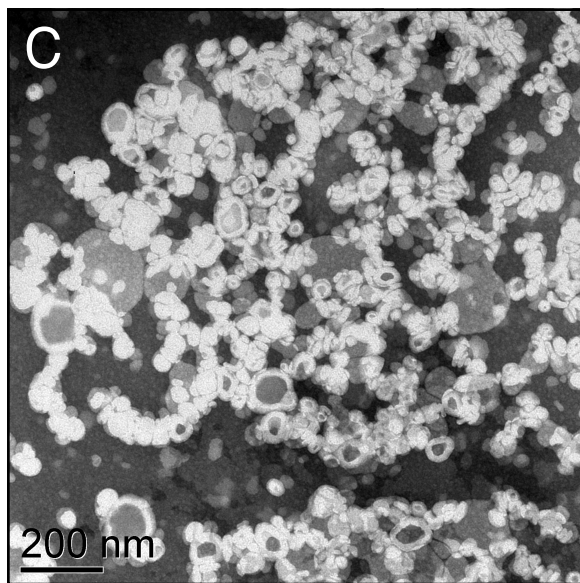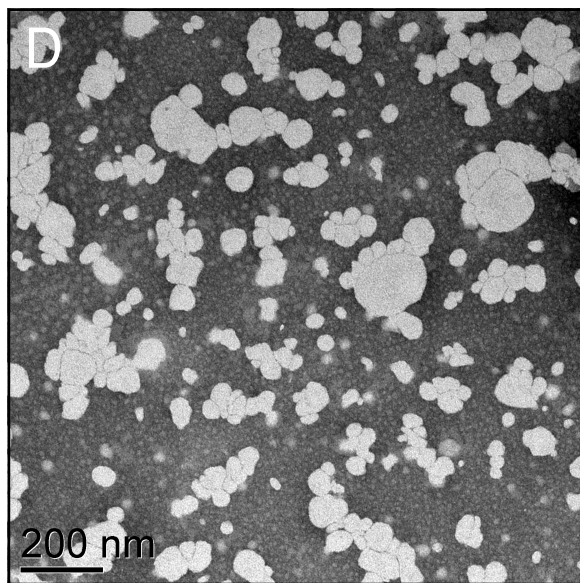

Supplement: FIG S7 [file mbo003173316sf7.pdf]
