# Supplementary material for: Mutations That Alter the Bacterial Cell Envelope Increase Lipid Production
Source: mBio. 2017 May 23;8(3):e00513-17. doi: 10.1128/mBio.00513-17 (PMC5442454; doi:10.1128/mBio.00513-17)
Supplement: TABLE S1 [file mbo003173316st1.pdf]

Table S1. Strains and plasmids used in this study

Strain	Relevant properties	Source or Reference
E. coli		
DH5α	Host for cloning and plasmid amplification	Bethesda Research Laboratories
DH5α/λ*pir*	Host for cloning Tn5 insertion sites; Φ80*dlacZ*ΔM15 Δ(*lacZYA-argF*) U169 *recA1 hsdR17 deoR thi-1 supE44 gyrA96 relA1/λpir*	(70)
S17-1	Donor for conjugation; C600∷RP-4 2-(Tc∷Mu) (Kn∷Tn7) *thi pro hsdR recA* Tra^+	(71)
BW20767	Donor for conjugation; RP-2-Tc::Mu-1 kan::Tn7 integrant *leu-63*::IS10 *recA1 zbf-5 creB510 hsdR17 endA1 thi uidA* (Δ*Mlu*I)::*pir+*	(72)
R. sphaeroides		
2.4.1	Wild-type strain	(73)
Δ0382 (parent strain)	ΔRSP0382 in 2.4.1	(20)
HLM01	Δ0382 with Tn5 insertion at Chr1: 1,471,645; Km^R	This study
HLM02	Δ0382 with Tn5 insertion at Chr1: 1,469,665; Km^R	This study
HLM03	Δ0382 with Tn5 insertion at Chr2: 274,987; Km^R	This study
HLM04	Δ0382 with Tn5 insertion at Chr1: 2,814,885; Km^R	This study
HLM05	Δ0382 with Tn5 insertion at Chr1: 2,970,757; Km^R	This study
HLM06	Δ0382 with Tn5 insertion at Chr2: 938,456; Km^R	This study
HLM07	Δ0382 with Tn5 insertion at Chr1: 2,086,261; Km^R	This study
HLM08	Δ0382 with Tn5 insertion at Chr1: 1,189,239; Km^R	This study
HLM08b	Same as HLM08, isolated independently from screen	This study
HLM09	Δ0382 with Tn5 insertion at Chr1: 1,395,725; Km^R	This study
HLM10	Δ0382 with Tn5 insertion at Chr1: 916,649; Km^R	This study
KL116	HLM02 carrying plasmid pKCL22 to express Myc-tagged NtrX; Km^R, Sp^R	This study
ΔNrtYXΔ0382	ΔRSP2839, ΔRSP2840, ΔRSP0382 in 2.4.1	This study
ΔChrRΔNrtYXΔ0382	Δ*chrR-1*::*dfr,* ΔRSP2839, ΔRSP2840, ΔRSP0382 in 2.4.1.	This study
**Plasmids**		
pRL27	Tn5-RL27 delivery vector (Km^R-*ori*R6 K)	(61)
pK18mobsacb	Broad host range mobilizable vector; Km^R *oriV oriT mob sacB*	(68)
pKCL20	5,939-bp *R. sphaeroides* genomic region containing RSP2839 and RSP2840 cloned into the XbaI and HindIII sites of pK18mobsacB; Km^R	This study
pKCL21	pKCL20 with RSP2839 and RSP2840 deleted; Km^R	This study
pJDN27	pSUP202-derived suicide plasmid that creates an insertion of Tp^R gene into a deletion of *chrR* gene	(69)
pIND5-myc	Expression vector with IPTG-inducible promoter, includes 3X myc tag; Sp^R	(74)
pKCL22	N-terminally 3X myc tagged *ntrX* (RSP 2840) cloned into BamHI and HindIII sites of pIND5-myc; Sp^R	This study
