# Supplementary material for: Mutations That Alter the Bacterial Cell Envelope Increase Lipid Production
Source: mBio. 2017 May 23;8(3):e00513-17. doi: 10.1128/mBio.00513-17 (PMC5442454; doi:10.1128/mBio.00513-17)
Supplement: TABLE S3 [file mbo003173316st3.pdf]

Table S3. Relative fatty acid content of *R. sphaeroides* ΔChrRΔNtrYXpd.

Strain	C16:1	C16:0	C18:1	C18:0	19M-UFA	19Fu-FA
**Cells**	3.2 (0.8)	11.3 (0.8)	69.2 (2.2)	12.5 (0.7)	0.8 (0.6)	2.9 (0.5)
**Media**	0.9 (0.8)	13.2 (2.1)	64.5 (6.6)	15.9 (3.0)	1.3 (0.3)	4.2 (1.8)

Percentage composition of the individual fatty acid species. 11-methyl-octadecanoate (19M-UFA), 10,13-epoxy-11-methyl-octadecadienoic acid (19Fu-FA). Standard deviation in parentheses. N=7.
